# Preparation of BMP-2/PDA-BCP Bioceramic Scaffold by DLP 3D Printing and its Ability for Inducing Continuous Bone Formation

**DOI:** 10.3389/fbioe.2022.854693

**Published:** 2022-04-06

**Authors:** Ziyang Yang, Li Xie, Boqing Zhang, Gang Zhang, Fangjun Huo, Changchun Zhou, Xi Liang, Yujiang Fan, Weidong Tian, Yinghui Tan

**Affiliations:** ^1^ Department of Stomatology, Xinqiao Hospital, Third Military Medical University, Chongqing, China; ^2^ State Key Laboratory of Oral Diseases and National Clinical Research Center for Oral Diseases and Engineering Research Center of Oral Translational Medicine, Ministry of Education and National Engineering Laboratory for Oral Regenerative Medicine, West China Hospital of Stomatology, Sichuan University, Chengdu, China; ^3^ National Engineering Research Center for Biomaterials, Sichuan University, Chengdu, China

**Keywords:** bone regeneration, DLP 3D printing, biphasic calcium phosphate, polydopamine, bone morphogenetic protein-2 (BMP-2)

## Abstract

Digital light processing (DLP)-based 3D printing is suitable to fabricate bone scaffolds with small size and high precision. However, the published literature mainly deals with the fabrication procedure and parameters of DLP printed bioceramic scaffold, but lacks the subsequent systematic biological evaluations for bone regeneration application. In this work, a biphasic calcium phosphate (BCP) macroporous scaffold was constructed by DLP-based 3D printing technique. Furthermore, bone morphogenetic protein-2 (BMP-2) was facilely incorporated into this scaffold through a facile polydopamine (PDA) modification process. The resultant scaffold presents an interconnected porous structure with pore size of ∼570 μm, compressive strength (∼3.6 MPa), and the self-assembly Ca-P/PDA nanocoating exhibited excellent sustained-release property for BMP-2. Notably, this BMP-2/PDA-BCP scaffold presents favorable effects on the adhesion, proliferation, osteogenic differentiation, and mineralization of bone marrow stromal cells (BMSCs). Furthermore, *in vivo* experiments conducted on rats demonstrated that the scaffolds could induce cell layer aggregation adjacent to the scaffolds and continuous new bone generation within the scaffold. Collectively, this work demonstrated that the BMP-2/PDA-BCP scaffold is of immense potential to treat small craniofacial bone defects in demand of high accuracy.

## Graphical Abstract

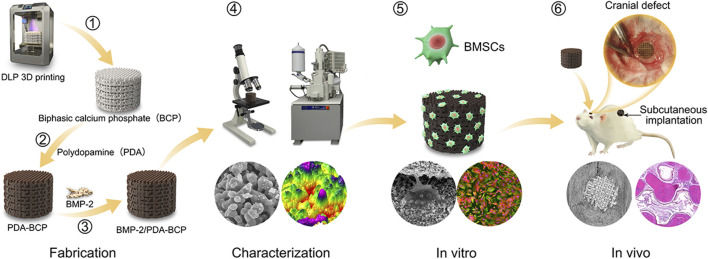

## 1 Introduction

Bone tissue defects caused by trauma, infection, cancer, congenital diseases, and other reasons are increasingly common worldwide, which will greatly affect normal life and life quality of the patients (Green et al., 2017; Aghali, 2021). Nowadays, commonly used bone grafts to treat bone defects include autografts (gold standard), allografts, and xenografts. However, autografts and allografts are both restricted by issues of donor tissue availability, donor site morbidity, and anatomic shape mismatch, while both allografts and xenografts present shortcomings with immune response complications, risk of disease transmission, and lack of osteogenetic properties (Panagopoulos et al., 2017; Shang et al., 2021). These issues have driven great research enthusiasm into new, cost-effective, and clinical-transformable strategies to treat critical-sized bone defects (Xie et al., 2021; Zhu et al., 2021).

With the advent of tissue engineering science and technology, tissue-engineered bone scaffolds are promising alternative choices for treatment of bone defects (Tan et al., 2021). Understandably, an ideal bone scaffold is expected to possess these properties (Ricciardi and Bostrom, 2013): 1) a match between the shape and size of the scaffold with the irregular and customized recipient site; 2) adequate porosity for new bone growth and new vessels; 3) mechanical properties consistent with the surrounding native bone; 4) cytocompatibility and biocompatibility of the scaffold; and 5) osteoconductivity and osteoinductivity of the scaffold materials. Therefore, a variety of biomaterials and manufacturing methods have been developed to fabricate patient-specific bone scaffolds in the past decades for repairing bone defects (Fu et al., 2021; Zhang et al., 2021; Zhao et al., 2021).

During the last decade, great progress has been achieved using 3D printing technologies to construct macroporous bioceramic scaffolds, thereby revolutionizing the traditional treatments of bone defects (Wen et al., 2017; Zhang L et al., 2019). Based on medical imaging data, 3D printing enables the mold-free fabrication of patient-specific bone substitutes with complex configuration directly from bioceramic powders and precise design of the structures at the macro- and micro-scales. Various 3D printing methods, including fused deposition modeling (FDM), selective laser sintering (SLS), stereolithography (SLA), and digital light processing (DLP)-based 3D printing, have been used for bone scaffold fabrication (Lee et al., 2018; Lin et al., 2019; Charbonnier et al., 2021). Compared with other 3D printing techniques, the DLP-based 3D printing has prominent advantages of high print resolution and fast print speed (Hong et al., 2020), which is suitable for printing scaffolds with small size and high precision. In regard of some bone defects in specific anatomic sites, e.g., cranial and maxillofacial bones, bone scaffold with small-size, shape-irregularity, and high-precision should be used, to recover a high-standard of functional or aesthetic appearance (Shen et al., 2020). In these cases, DLP-based 3D printing holds high promise.

Nowadays, DLP-based 3D printing is widely used to fabricate hydrogel scaffolds (Gong et al., 2020) and there are comparatively less reports to fabricate bioceramic scaffolds. For bioceramic scaffold fabrication, bioceramic powders should first be prepared in slurry type combined with photocurable resin as 3D printing inks (Kim et al., 2020). After DLP-based 3D printing, the scaffold subsequently should be thermally processed to remove the organic resin and obtain the final bioceramic scaffold. There are several reports regarding preparing DLP-printed bone scaffolds using hydroxyapatite (HA), *ß*-tricalcium phosphate (*β*-TCP), and calcium silicate bioceramics (Shao et al., 2017; Zhang J et al., 2019; Schmidleithner et al., 2019). Zhang et al. (2020) recently prepared a structurally diversified Haversian bone-mimicking scaffold via DLP-based 3D printing using bioceramic Ca2MgSi2O7, which is hard to fabricate through the FDM printing method. However, the existing published reports mainly dealt with the fabrication process and parameters of DLP printed bioceramic scaffold, but lack the subsequent systematic biological evaluations for bone regeneration application (Li et al., 2021). Recently, there is a growing interest in developing HA/β-TCP biphasic calcium phosphate (BCP) bioceramics as bone scaffolding materials because they are more effective in bone regeneration than pure HA or pure *ß*-TCP, and have a controllable degradation rate (Kim and Park, 2020). However, there is rarely published literature reporting DLP-based printed BCP scaffolds in bone tissue engineering applications.

On the other hand, not only the customized shape and interconnected pores but hierarchical porous structures and biological properties (both osteoconductivity and osteoinductivity) of bone scaffolds are critical factors affecting cell behavior and osteogenic performance (Feng et al., 2021). Compared to scaffolds with a smooth surface, scaffolds with a porous surface structure in micro/nano-sized scale were found to be beneficial (Zhu et al., 2017). In addition, bone morphogenetic proteins (BMPs), particularly BMP-2, are capable of inducing the osteogenic differentiation of mesenchymal stem cells (MSCs) and accelerating bone regeneration in the clinic, showing excellent osteoinductivity (Zhou et al., 2018). However, the control over the surface morphology of the scaffold struts and the *in situ* delivery of bioactive agents appear difficult directly through DLP 3D printing, which calls for subsequent surface modification treatment.

Owing to the satisfactory biocompatibility, biodegradability, and substrate independent, mussel-inspired polydopamine (PDA) modification has great potential for further surface modification of 3D porous scaffolds (Liu et al., 2014). Ma et al. (2016) reported that a uniformly self-assembled Ca-P/PDA nanolayer was deposited on a Ca7Si2P2O16 bioceramic scaffold fabricated by FDM printing. Furthermore, the formed self-assembled Ca-P/PDA composite nanolayer significantly enhances the attachment, proliferation, alkaline phosphate (ALP) activity, and bone-related gene expression of rabbit bone mesenchymal stem cells (rBMSCs). Besides, self-assembled PDA coating can also act as growth factors (GFs) delivery vehicle, which can adhere strongly to almost every kind of biomolecule owing to copious catechol moieties (Ryu et al., 2018). As far as we know, DLP-based printed BCP bioceramic scaffold with a GF-delivery PDA nanocoating has not been reported yet.

**FIGURE 1:**
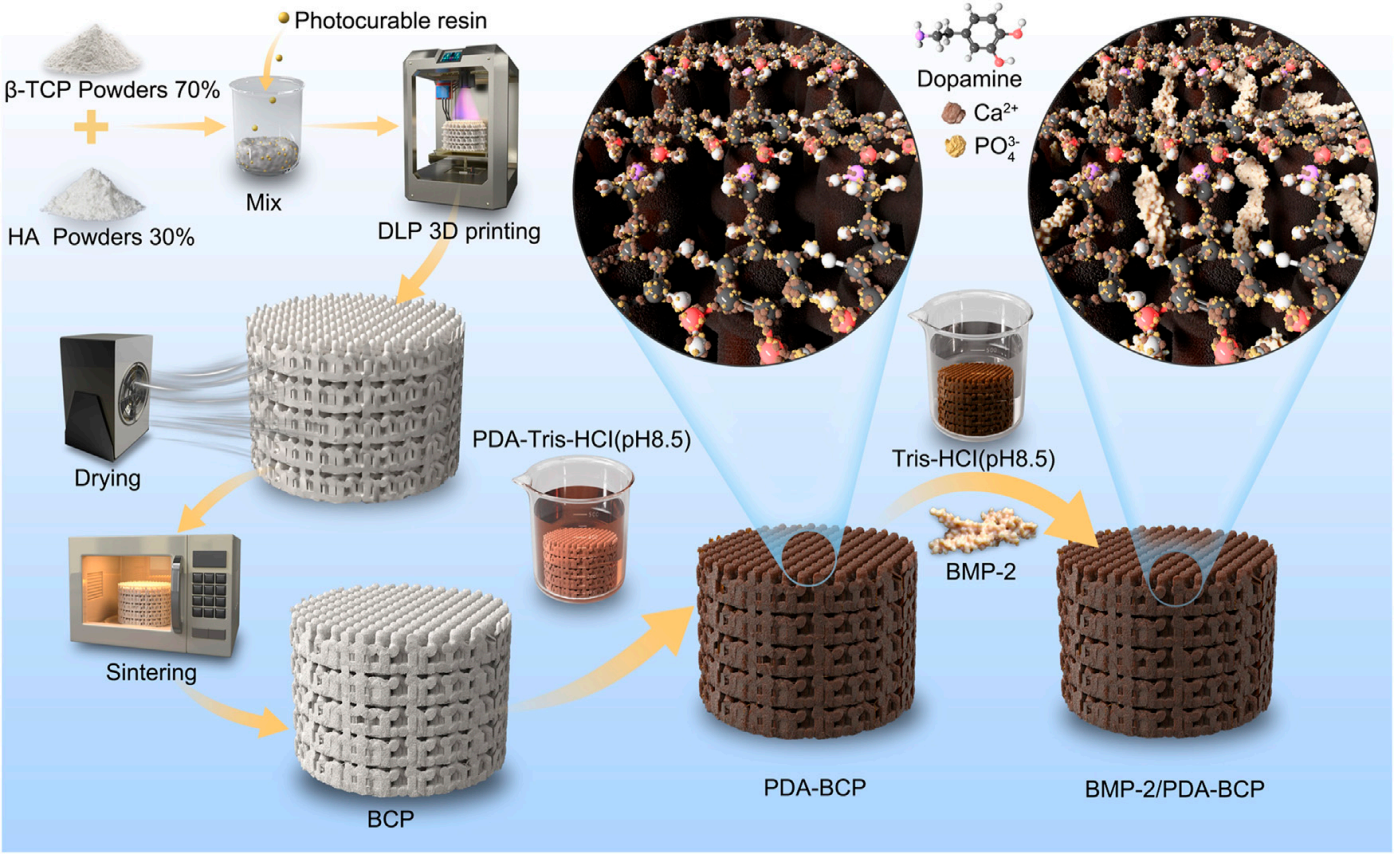
Schematic diagram of DLP 3D printing, modification of PDA, and grafting of BMP-2. DLP: digital light processing; PDA: polydopamine; BMP-2: bone morphogenetic protein-2.

Based on the above background, we reported the fabrication of a DLP-based 3D printed BCP bioceramic scaffold with self-assembly PDA modification and BMP-2 incorporation. Collectively, we proposed a novel bone tissue scaffold with comprehensive properties including designable macroporous structures, suitable bioceramic substrates, and osteogenic ability. The physicochemical properties of the PDA-modified scaffold, including surface topography, surface roughness, hydrophilicity, and mechanical strength are characterized. Also, the sustained release behavior of BMP-2 on the PDA-BCP scaffold was assessed. A schematic diagram of the fabrication procedure of porous scaffolds is shown in Figure 1. Then, the cell attachment, viability, proliferation, and osteogenic differentiation of BMSCs were systematically evaluated. We finally evaluated its bone regeneration capacity in a rat cranial bone defect model via micro-CT and histomorphometric analysis.

## 2 Materials and Methods

### 2.1 Fabrication of the DLP-Based 3D Printed BCP Scaffold

The 3D structures of the scaffolds were designed by Materialize 3-matic software to obtain the 3D stereolithography (STL) files. With a mass ratio of 7:3, *ß*-tricalcium phosphate (*β*-TCP) powders and hydroxyapatite (HA) powders were mixed and ball milled with a mass fraction of 25% photosensitive resin to obtain the precursor slurry. The bioceramic powders were supplied by the Engineering Research Center for Biomaterials of Sichuan University.

The precursor slurry was printed and cross-linked by a DLP-based 3D printer (AdMaflex 130Plus, AdMatec, Netherlands) following the STL files. The exposure time was 300 ms and the single-layer scanning time was 20 s for each 50-μm thick slice. The wavelength of the light source was 400 nm. After printing, the samples were detached from the prototyping platform and washed by ultrasonication and further washed in alcohol for 15 min to remove the uncured resin and the solid bottom of the scaffolds were then removed. The green bodies of the BCP scaffolds were then sintered in a muffle furnace to obtain the pure bioceramic scaffolds. The sintering procedure was described as follows: first heated to 800°C at a rate of 5°C/min and kept for 2.7 h, then heated to 1100°C at a rate of 5°C/min for 5 h, and finally cooled to room temperature naturally in the furnace. Porous scaffolds with the preset pore sizes of 300 μm, 600 μm, 800 μm, and 1000 μm were fabricated. The size of each printed scaffold before and after sintering was measured by vernier caliper and the shrinkage rates were calculated.

### 2.2 Preparation and Characterization of PDA-Coated BCP Scaffolds

To prepare a self-assembled Ca-P/PDA nanolayer on the surface of the BCP bioceramic scaffold, dopamine hydrochloride (Aladdin, China) was first dissolved in 10 mM Tris-HCl buffer (pH 8.5) at a concentration of 2 mg/ml, 4 mg/ml, and 8 mg/ml, respectively. BCP scaffolds were soaked in Tris-dopamine solution for 24 h at 37°C in a 100-rpm oscillator. The samples were denoted as 2PDA-BCP, 4PDA-BCP, and 8PDA-BCP, respectively. After soaking, the samples were rinsed in ultrapure water three times and dried at 60°C overnight.

The gross images, surface microstructure, surface chemical composition, and surface roughness of the bare BCP scaffold and PDA-BCP scaffolds were characterized by stereoscopic microscope (SZX2, Olympus, Japan), scanning electron microscopy (SEM, SU8220, Hitachi, Japan), atomic force microscopy (AFM, Dimension Icon, Bruker, Germany), energy-dispersive spectroscopy (EDS, SU8220, Hitachi, Japan), and Fourier transform infrared spectroscopy (FTIR, Nicolet iN10, ThermoFisher, Massachusetts, United States). The hydrophilicity of scaffolds was detected by investigating with a water contact angle tester (DSA30, KRÜSS, Germany).

The released calcium (Ca) and phosphorus (P) ionic concentrations from the scaffolds were examined by inductively coupled plasma optical emission spectroscopy (ICP-OES). The scaffolds were soaked in the Tris-HCl solution (pH = 7.4) with a mass/volume ratio of 50 mg/ml at 37°C under constant agitation at 100 rpm. After 1, 3, and 7 d, the supernatants were collected for detection.

The compressive strength of the scaffolds was assessed by applying a vertical load on the samples using a universal testing machine (UTM, AGS-X, Shimadzu, Japan), all tests were implemented at a strain rate of 1 mm/min. The stiffness of specimens was obtained from the curve.

### 2.3 BMP-2 Loading and Release Profile Detection

The scaffolds were sterilized by epoxy ethane in advance. BMP-2 solution was prepared by dissolving the BMP-2 (Human BMP2 Protein [Recombinant His], LSBio, United States) in 10 mM Tris-HCl buffer (pH 8.5). Each of the scaffolds (BCP, 2PDA-BCP, 4PDA-BCP, and 8PDA-BCP) was immersed in 250 ng/ml BMP-2 solution for 24, 48, and 72 h, respectively. After that, the supernatants were collected, and the amount of unattached BMP-2 was quantified by ELISA assay using a human bone morphogenetic protein two ELISA kit according to the manufacturer’s instructions (Jingmei Biotechnology, China). The adsorbed BMP-2 on the scaffolds was then calculated.

To investigate the *in vitro* release profiles of BMP-2 from BCP and PDA-BCP scaffolds, each scaffold was immersed in 1 ml of 250 ng/ml BMP-2 solution and incubated for 48 h under aseptic conditions at 37°C. Then, the scaffolds were washed gently with PBS and stored at −20°C for later use. Then they were incubated in 500 μL PBS at 37°C in an incubator. The total release medium was taken out and frozen at -80°C at Day 1, 3, 5, 7, 10, 14, 21, 28, and 35, and replaced with an equal amount of fresh PBS solution. The quantitative measurement of the BMP-2 in the supernatants was performed using an ELISA Kit as mentioned above. The release curve was calculated in terms of the cumulative release percentage of BMP-2 (%) with incubation time. A standard curve was generated using known concentration (0.25–8 ng/ml).

### 2.4 *In vitro* Cell Responses of BMSCs on Scaffolds

#### 2.4.1 Isolation and Culture of BMSCs

All the procedures were under a protocol approved by the Ethics Committee, West China School of Stomatology, Sichuan University, China. BMSCs were isolated from long bones of SD rats according to the previous literature (Boregowda et al., 2016). The cells were cultured in 90% *α*-minimum essential medium (*α*-MEM; HyClone) containing 10% fetal bovine serum (Gibco, a product line of Thermo Fisher Scientific, Waltham, MA, United States), 100 IU/ml penicillin, and 100 μg/ml streptomycin. The medium was changed every 3 d and adherent cells were passaged until 80–90% confluence was achieved. Third-passage cells were used for subsequent experiments.

#### 2.4.2 Cellular Viability, Proliferation, and Apoptosis of BMSCs on Scaffolds

The qualitative assessment on the cell viability of BMSCs was evaluated using the Live/Dead assay kit (keyGEN bioTECH, Jiangsu, China) after being cultured for 1 d and 4 d. In brief, a Live/Dead staining solution contained 2 μM Calcein AM and 8 μM propidium iodide (PI) was prepared in PBS, with Calcein AM detecting live cell and PI for dead cell identification. The samples were incubated with the staining solution at 37°C for 30 min, then they were washed again with PBS three times, and stained cells were imaged under a confocal laser scanning microscope (CLSM, Olympus FV1200, Olympus Corporation, Japan). Live cell numbers were measured in three randomly selected images of each sample using NIH ImageJ 1.52i software.

On the hand, BMSCs were seeded on scaffolds in 48-well plates at a density of 1 × 104 cells/well and cultured for 1, 4, and 7 d. At each time point, 40 μl of CCK-8 solution mixed with 400 μl culture medium was added to each well and the plates were incubated for 2.5 h. Then, 110 μl aliquot was taken from each well and transferred to a fresh 96-well plate. The absorbance values were read at 450 nm (*n* = 3) using a microplate reader (Spectrophotometer; ThermoFisher, Massachusetts, United States).

For the apoptosis assay, the scaffolds were placed to the upper chamber of the transwell and nearly 1 × 105 cells/well were cultured on the lower chamber for 7 d. The cells were trypsinized and the cell suspension was centrifuged. BMSCs seeded on culture dishes without scaffolds were prepared as a blank control. The cells were stained using an Annexin V-FITC/PI Apoptosis assay Kit (Absin, Shanghai, China) following the manufacturer’s instructions and analyzed using a BD Accuri C6 Flow Cytometer (*n* = 3).

#### 2.4.3 Cell Adhesion on Scaffolds

For the evaluation of cell attachment, BMSCs with a density of 1 × 105 cells/well were seeded on BCP, PDA-BCP, BMP-2-BCP, and BMP-2/PDA-BCP scaffolds. After cultured for 1 d, the scaffolds were rinsed with PBS three times and fixed with 4% paraformaldehyde for 4 h. Then the fixative was removed by washing with PBS, followed by sequential dehydration in graded ethanol. The specimens were dried in hexamethyldisilane (HMDS) for 30 min before coating with gold for SEM analysis. The morphology of the attached cells was observed under SEM.

Meanwhile, cells cultured on scaffolds for 7 d were stained and observed under CLSM. The fixed cells were stained with phalloidin 647 Conjugate (1:1,000, Absin) for F-actin and DAPI (1:1,000, Sigma) for cell nuclei. Three random images were selected for measurement of mean gray value using ImageJ software.

### 2.5 Effect of Scaffolds on the Osteogenic Differentiation of BMSCs

#### 2.5.1 ALP Activity and ALP Staining of BMSCs on Scaffolds

The ALP activity assay and ALP staining were performed to evaluate the osteogenic differentiation of BMSCs. There were 5 × 104 cells seeded onto each scaffold and placed in a 48 well plate and cultured for 4, 7, and 14 d. After cell lysis, the supernatant was centrifuged and used for the ALP kit detection (Beyotime, Shanghai, China) according to the manufacturer’s instruction The results were normalized to the total protein content, which was measured by BCA protein assay kit (KeyGen, China).

For ALP staining, BMSCs with a density of 5 × 104 cells/well were seeded on each pristine well (transwell of 12 wells). After 24 h, the medium was exchanged with osteogenic medium. ALP staining was evaluated after incubation for 10 d and performed by an ALP staining kit (Beyotime biotechnology, Shanghai, China). Briefly, the cell-seeded wells were fixed with 4% paraformaldehyde at room temperature for 15 min. After washing with PBS carefully, 5-Bromo-4-Chloro-3-Indolyl-Phosphate/Nitro-Blue-Tetrazolium (BCIP/NBT) staining solution was added to ensure that the sample was fully covered. The samples were incubated for 30 min in the dark at room temperature. The reaction was terminated after washing with PBS 1–2 times, and the cell staining images were observed under an inverted microscope (Olympus, Tokyo, Japan).

#### 2.5.2 Calcium Deposition and Mineralization Detection

The effect of the scaffolds on extracellular mineralization was detected on Day 21 of osteogenic induction. In short, medium was removed, gently washed with PBS 3 times, and fixed with 4% paraformaldehyde at room temperature for 15 min. After being washed with deionized water 3 times, 0.2% alizarin red solution was added for 30 min at room temperature. The plates were washed by deionized water 3 times, images were taken with digital cameras (EOS550D, Canon, Japan). In addition, the calcium nodules area was conducted in three random fields using ImageJ software. After the images were collected, 10% cetylpyridinium chloride (Sangon Biotech, Shanghai, China) (1 g with 10 m LDD water, shaken well in a shaker at 37°C to form a transparent solution) was incubated in the dark for 30 min, and 100 μl was transferred to a 96-well plate to test the OD value at 560 nm.

#### 2.5.3 Osteogenic-Related Gene Expression

The osteogenic-related mRNA expression of BMSCs, including runt-related transcription factor 2 (Runx-2), osteocalcin (OCN), collagen type 1 (Col-1), and osteopontin (OPN), on the scaffolds was determined by real-time quantitative PCR. Cells with density of 1 × 106 cells/well were seeded onto each scaffold, which was placed in 6-well plates. The cells were incubated in osteogenic induction medium for 4, 7, and 10 d. The osteogenic medium contained 10 mM *ß*-glycerol phosphate, 0.2 mM ascorbic acid, 0.01 μM 1,25-dihydroxy vitamin D3, 10-8 M dexamethasone. After the preset time point, Trizol Reagent^®^ (Invitrogen Pty Ltd., Australia) solution was used to extract the total RNA according to the manufacturer’s instructions. Relative expression levels for each gene were normalized against the cycle threshold (Ct) value of the house keeping gene (GAPDH) and determined by using the delta Ct (ΔCt) method. Each sample was performed in triplicate. The primer sequences used are described in Table 1.

**TABLE 1:** List of qRT-PCR primers.

Target cDNA	Primer sequence (5′-3′)
GAPDH-Forward	GAC​ATG​CCG​CCT​GGA​GAA​AC
GAPDH-Reverse	AGC​CCA​GGA​TGC​CCT​TTA​GT
Runx2- Forward	CTT​CGT​CAG​CGT​CCT​ATC​AGT​TCC
Runx2-Reverse	TCC​ATC​AGC​GTC​AAC​ACC​ATC​ATT​C
OCN- Forward	ACT​CTG​AGT​CTG​ACA​AAG​CCT​TCA​TG
OCN-Reverse	GGC​TCC​AAG​TCC​ATT​GTT​GAG​GTA​G
COL 1- Forward	CGA​GTC​ACA​CCG​GAA​CTT​GG
COL 1-Reverse	CCA​ATG​TCC​AAG​GGA​GCC​AC
OPN- Forward	AAC​ACT​CAG​ATG​CTG​TAG​CCA​CTT​G
OPN-Reverse	GCT​TTC​ATT​GGA​GTT​GCT​TGG​AAG​AG

#### 2.5.4 Immunofluorescence Images for Osteocalcin and Osteopontin Expression

BMSCs were cultured in osteogenic medium in the presence of each scaffold as described above. After 14 days, cells were fixed with 4% paraformaldehyde solution, washed with PBS, and stored at 4°C until cytochemistry labeling. Cells were permeabilized with 0.2% (v/v) Triton X-100 for 20 min and nonspecific binding blocked with a 5% BSA solution (Solarbio, China). Then they were incubated overnight at 4°C with the primary antibodies osteocalcin (GTX13418, GeneTex) and osteopontin (AF0227, Affinity), respectively. Then, the cells were washed with PBS and incubated with appropriate Fluor-coupled secondary antibodies for 1 h. Nuclei were counter-stained with DAPI. The cells were thoroughly washed with PBS before observation under CLSM.

### 2.6 Animal Experiments

#### 2.6.1 Animal Surgery Procedure

Eight-week-old male SD rats with a weight of 280 ± 20 g were obtained from the Sichuan University Animal Center (Sichuan, China). All procedures concerning animal use were under a protocol approved by the Research Ethics Committee, West China Hospital of Stomatology of Sichuan University. Briefly, the animals were anesthetized by an intraperitoneal injection of 3% pentobarbital (1 ml/kg). Subsequently, rats were randomly divided into five groups: 1) BCP scaffold, 2) PDA-BCP scaffold, 3) BMP-2-BCP scaffold, 4) BMP-2/PDA-BCP scaffold, and 5) Control (without any scaffold). The scaffolds were immersed in 1 ml of 2500 ng/ml BMP-2 solution and incubated for 48 h under aseptic conditions at 37°C for the *in vivo* experiments. The scaffolds were subcutaneously implanted at the dorsum area of the SD rats for 1 and 4 weeks to study the *in vivo* biocompatibility and possible ectopic osteogenic ability. For the critical defect bone regeneration evaluation, a full-thickness calvarial bone defect (diameter: 8 mm) was created using a slow-speed dental drill. These rats were sacrificed under general anesthesia after 4, 8, and 12 weeks of implantation. The specimens, containing the cranial defect and 3–5 mm peripheral cortical bone adjacent to the defect, were harvested and fixed in 4% paraformaldehyde for further micro-computed tomography (micro-CT) and histological analysis.

#### 2.6.2 Micro-CT Imaging and Osteogenic Analysis

New bone formation was examined using high-resolution micro-CT (SkyScan 1176 desktop X-ray micro-CT system, Bruker, Billerica, MA, United States). The voltage was set to 80 kV and the current was 300 μA, with aluminum and cuprum filtration. 3D views of the region of interest were reconstructed by NRecon cone beam reconstruction software (Skyscan Company), and the threshold was optimized to isolate bone tissues and scaffolds. A cylinder area of 8 mm in diameter and 1.5 mm in height of the skull containing scaffolds was selected as the region of interest. The ratio of bone volume to total volume (BV/TV) and number of bone trabeculae (Tb.N) in the bone defects were calculated by using the auxiliary histomorphometric software (Ctan, Belgium).

#### 2.6.3 Histological and Immunohistochemical Analysis

After micro-CT analysis, the harvested samples were fixed in 4% paraformaldehyde for 2 weeks and then were decalcified using 10% EDTA solution (pH 7.4) at 4°C for 4 weeks 5-μm thick sections were prepared and stained with hematoxylin and eosin (H&E) or Masson’s trichrome according to the manufacturers’ instructions. The sections were observed using a Panoramic MIDI Ⅱ pathological section scanner (3DHISTECH, Ltd., Budapest, Hungary). At least five slices of Masson’s trichrome stain for each group were analyzed. The area ratios of new bone were calculated in the NIH ImageJ 1.52i software. Immunohistochemistry (IHC) of OCN was performed to evaluate bone matrix deposition.

### 2.7 Statistical Analysis

Statistical analysis was conducted using GraphPad Prism 9.0.0 Software (GraphPad Software Inc.). All data were expressed as means ± standard deviations (SD) and were analyzed using two-way ANOVA followed by a Turkey HSD post-hoc test, differences with *p*-values (*p < 0.05, **p < 0.01, ***p < 0.001) were considered statistically significant. All quantifications were done with ImageJ 1.52 software on high resolution images.

## 3 Results

### 3.1 Fabrication and Characterization of PDA-Modified 3D-Printed BCP Scaffolds

#### 3.1.1 Fabrication and Microstructure of DLP-Printed BCP Scaffold

**FIGURE 2:**
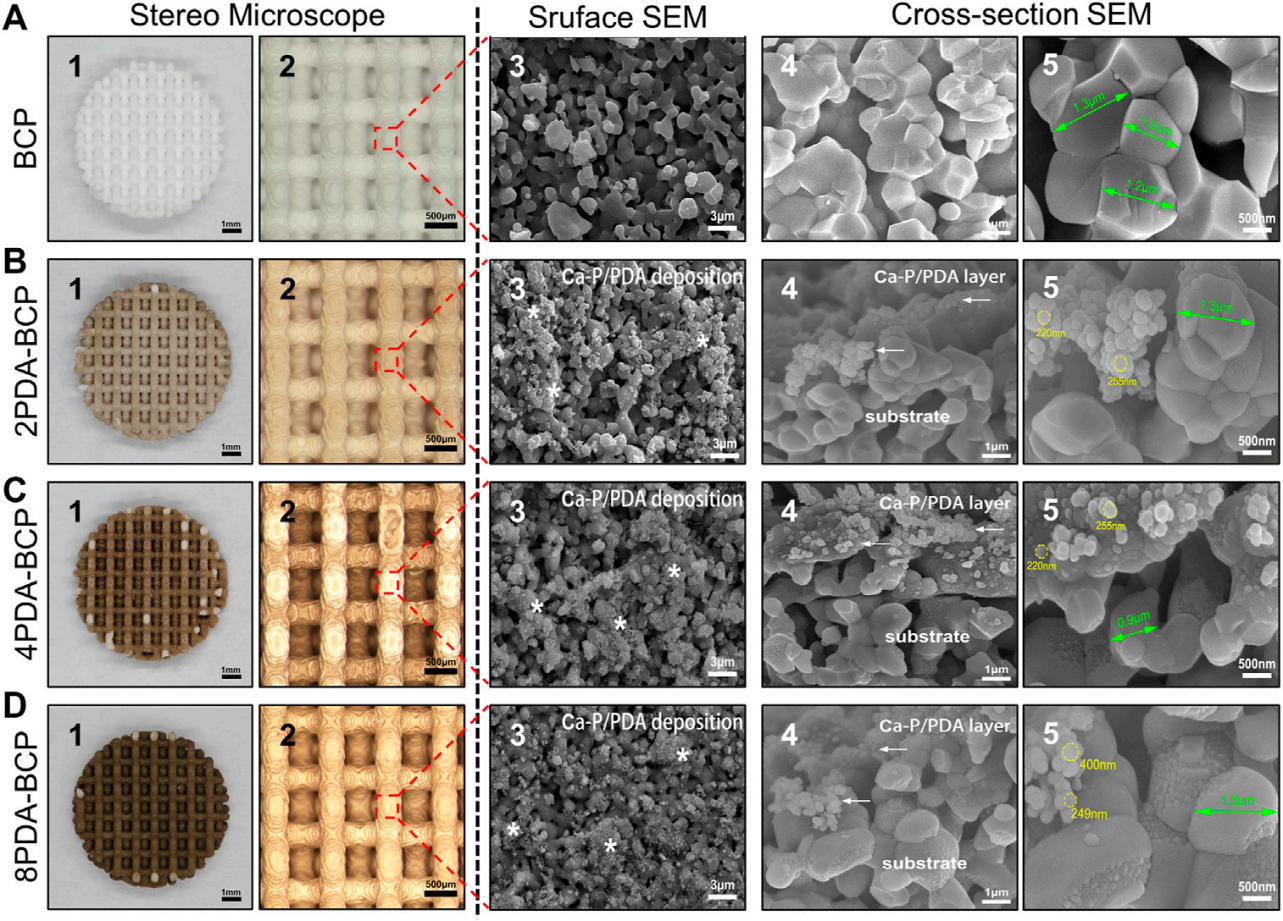
Macroscopic morphology, surface microstructures, and cross-section morphologies of 3D printed scaffolds and PDA modification scaffolds on stereo microscope and scanning electron microscope. **(A)** BCP. **(B)** 2PDA-BCP. **(C)** 4PDA-BCP. **(D)** 8PDA-BCP. The white stars indicate deposition composed of Ca-P/PDA, the white arrows indicate the PDA layer and amorphous Ca-P nanoparticles. The grain size of BCP (green arrows), the size of new formation amorphous Ca-P nanoparticles (yellow circle). Scale bars = 1 mm, 500 μm, 3 μm, 1 μm, 500 nm.

BCP scaffolds with different designed pore sizes (300, 600, 800, 1000 μm) could be fabricated through DLP-based 3D printing technology and a followed sinter treatment (Supplementary Figure S1A). The shrinkage rates after sintering were about 17–21% (Supplementary Figures S1B, C). Scaffolds with designed pore size of 600 μm were chosen in the following experiments. The 3D printed disc-shape BCP scaffold was 7.8 ± 0.2 mm in diameter, 1.5 ± 0.1 mm in height (Supplementary Figure S1D). As shown in stereoscopic and SEM images (Figure 2A), the scaffold possessed a porous structure with inter-layered and regular 90–90° layer-by-layer pattern. The aperture was ∼570 ± 20 μm, the structure width was ∼500 μm, and the porosity was ∼67.3%. At high-magnification SEM images, the syncretic micro-scale crystal grains could be observed with size about 1.2 ± 0.3 μm.

#### 3.1.2 Preparation and Physicochemical Properties of PDA-BCP Scaffolds

The 3D-printed BCP scaffolds were then treated in DA-Tris-HCl solution with different concentrations (2, 4, and 8 mg/ml) for 24 h to obtain a self-assembly PDA layer on the scaffold surfaces. Optical stereo images (Figures 2B–D) showed that the PDA-BCP scaffolds turned from white to brown and the color became darker with the increase of the concentration. The SEM images of PDA-BCP scaffolds showed that newly formed spherical precipitations, size ranging from 200–400 nm, could be found on both the surfaces and cross-sectional surface, which were different from the pristine BCP crystallites. Besides, these newly formed crystals deposited on the scaffold surface can also be observed in the AFM images (Supplementary Figure S2). Also, FTIR spectra (Supplementary Figure S3) showed that all scaffolds exhibited vibrations of PO43-groups ranging from 960 to 1035 cm-1 ascribed to HA and TCP (Lee et al., 2017). For PDA-BCP scaffolds, FTIR peaks at 3379 cm-1 were corresponded to -OH and N-H groups and a range of characteristic peaks at 1500–1615 cm-1 were assigned to different N-H bands, which were identified from dopamine and polydopamine (Li et al., 2014). Furthermore, the EDS analysis showed that the Ca/P ratio on the bare BCP scaffold surface was 1.538, while the Ca/P ratios of the newly formed precipitates on PDA-BCP scaffolds ranged between 1.604 and 1.640 (Supplementary Figure S4). Collectively, these results suggested that a layer of calcium-phosphate (Ca-P)/polydopamine (PDA) was uniformly formed on the BCP scaffold (Feng et al., 2020).

**FIGURE 3:**
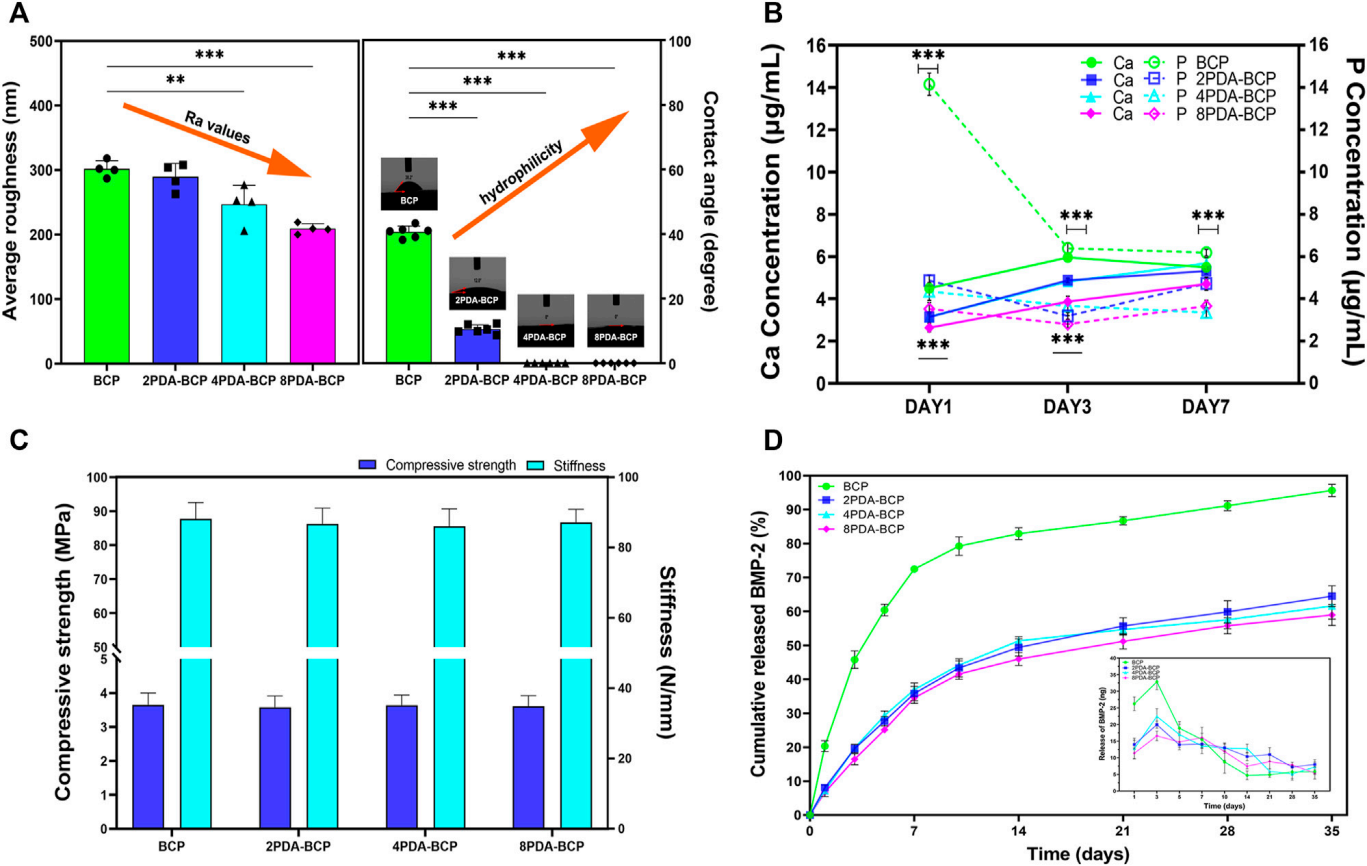
Characterization of BCP and PDA-BCP scaffolds. **(A)** The statistical analysis of average roughness and water contact angle for BCP, 2PDA-BCP, 4PDA-BCP, 8PDA-BCP scaffolds, respectively. **p < 0.01, ***p < 0.001. **(B)** The change of Ca and P ionic concentration from various scaffolds in the Tris–HCl solutions (pH 7.4), data are presented as the mean ± S.D. ***p < 0.001. **(C)** Compressive strength and stiffness of scaffolds. **(D)** Cumulative BMP-2 release kinetics from the BCP, 2PDA-BCP, 4PDA-BCP, and 8PDA-BCP scaffolds, the insert graph shows the release number of scaffolds in each time point.

The Ra values calculated from AFM data were 302 nm, 290 nm, 247 nm, and 209 nm for the BCP, 2PDA-BCP, 4PDA-BCP, and 8PDA-BCP respectively, indicating that the surface roughness of the scaffolds decreased gradually with the increase of PDA concentration (Figure 3A). The surface hydrophilicity of the BCP scaffold was improved after PDA modification (Figure 3A). The water contact angle (WCA) values of BCP and 2PDA-BCP scaffolds were 40.6 ± 1.8° and 10.5 ± 1.4°, respectively, while the 4PDA-BCP and 8PDA-BCP scaffolds were superhydrophilic with WCA of 0°. In addition, the ion solubility of the scaffolds is detected. As shown in Figure 3B, the concentrations of Ca2+ and PO43- ion dissolved from PDA-BCP scaffolds were significantly lower than that from the bare BCP scaffold. The average values of compressive strength and stiffness of the BCP and PDA-BCP scaffolds were about 3.6 MPa and 86 N/mm, respectively (Figure 3C). The results also indicated that PDA modifications have no adverse effect on the compressive strength and stiffness of the BCP scaffolds.

#### 3.1.3 BMP-2 Adsorption and Release Behavior

BMP-2 loading behaviors on BCP and PDA-BCP scaffolds were presented in Supplementary Figure S5. After 48 h, significantly more BMP-2 was immobilized (173.05 ng) on the 2PDA-BCP scaffold surfaces compared with that on the bare BCP scaffold (129.05 ng), while there is no significant difference among the three PDA-BCP scaffolds. The release kinetics of the BMP-2 from the scaffolds was investigated by ELISA assay. The release profiles of BMP-2 from the three PDA-BCP scaffolds exhibited similar trends and all showed a sustained release behavior with negligible burst release during the observed 35 d (Figure 3D). The cumulative released ratio of BMP-2 from the 2PDA-BCP scaffold was about 64.5% after 35 d. In contrast, the BMP-2 showed a burst release from the BCP scaffold within the initial 7 d. In the following experiments, 2PDA-BCP scaffold was chosen since it can meet the requirements for bone scaffold application as revealed by the above physicochemical and BMP-2 delivery properties.

### 3.2 *In vitro* Studies of PDA-Modified 3D-Printed BCP Scaffolds

#### 3.2.1 Cell Viability, Morphology, and Proliferation

**FIGURE 4:**
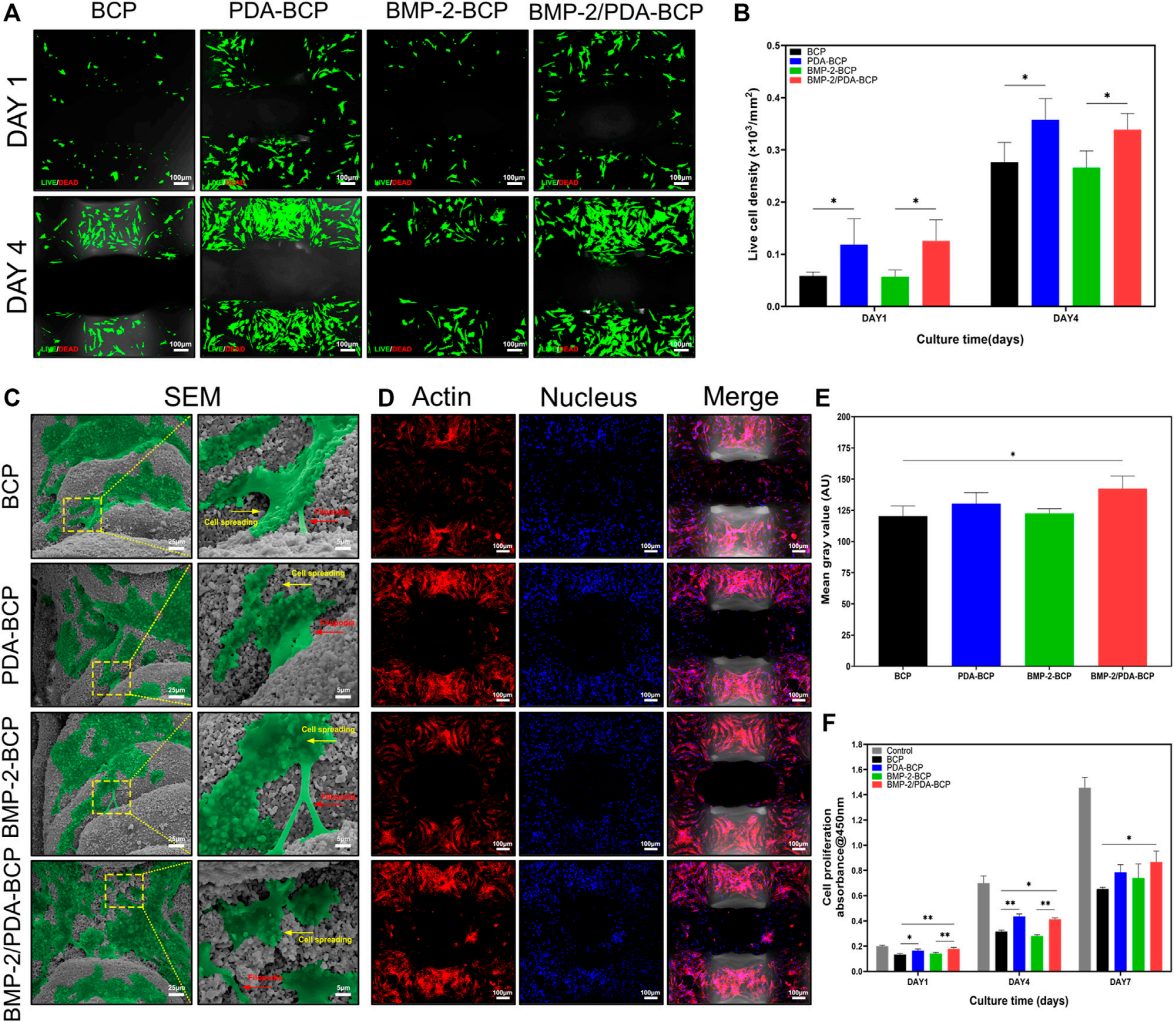
Cellular compatibility, adhesion, and proliferation of BMSCs on scaffolds. **(A)** Fluorescence images of live (green)/dead (red) cell staining and **(B)** live cell density of BMSCs grown on BCP, PDA-BCP, BMP-2-BCP, and BMP-2/PDA-BCP scaffolds after 1 and 4 d of culture. *p < 0.05. Scale bars = 100 μm. **(C)** Morphology of BMSCs adhered after 24 h on the surface of scaffolds. Scale bars = 25 μm. The higher magnification shows BMSCs with longer filopodial extension. Scale bars = 5 μm. **(D)** Confocal microscopic images of BMSCs attached to the internal struts of the scaffolds stained with F-actin (phalloidin, red) and nuclei (4′,6-diamidino-2-phenylindole, blue) at 7 d after cell seeding. Scale bars = 100 μm. **(E)** Quantitative evaluation of F-actin staining images. *p < 0.05. **(F)** CCK-8 assay for cell proliferation on various scaffolds for 1, 4, and 7 d culturation. The data are shown as the mean ± S.D. *p < 0.05, **p < 0.01.

The viability of BMSCs was qualitatively identified by live/dead fluorescent cell staining and observed by CLSM. As shown in Figure 4A, cells on all scaffolds displayed high viability, with almost no dead cells found in the images after 1 and 4 d of culture. In addition, the density of live cells on PDA-modified scaffolds was higher than that on bare BCP scaffolds (Figure 4B). Furthermore, the flow cytometry results (Supplementary Figures S6A, B) showed that the live cell ratios were all nearly 80% on the four kinds of scaffolds after incubation for 7 d, further demonstrating the excellent cell cytocompatibility of the scaffolds.

The adhesion and morphology of BMSCs on the scaffolds were further evaluated by SEM and CLSM (Figures 4C,D). After 24 h of culture, cells could be observed to adhere and spread well on all scaffolds, showing flattened polygonal shapes and pseudopodia extension. In addition, many spreading cells exhibited on all the scaffolds on Day 7 after seeding (Figure 4D). Cells on the BMP-2/PDA-BCP scaffold were significantly more than those in the bare BCP scaffold (Figure 4E). Consistently, the BMP-2/PDA-BCP scaffold exhibited a higher proliferation rate compared with the bare BCP scaffold (Figure 4F). Additionally, cells on the PDA-BCP scaffold seemed to proliferate faster compared with the bare BCP scaffold, although with no significant difference on Day 7.

#### 3.2.2 Osteogenic Differentiation and Mineralization

**FIGURE 5:**
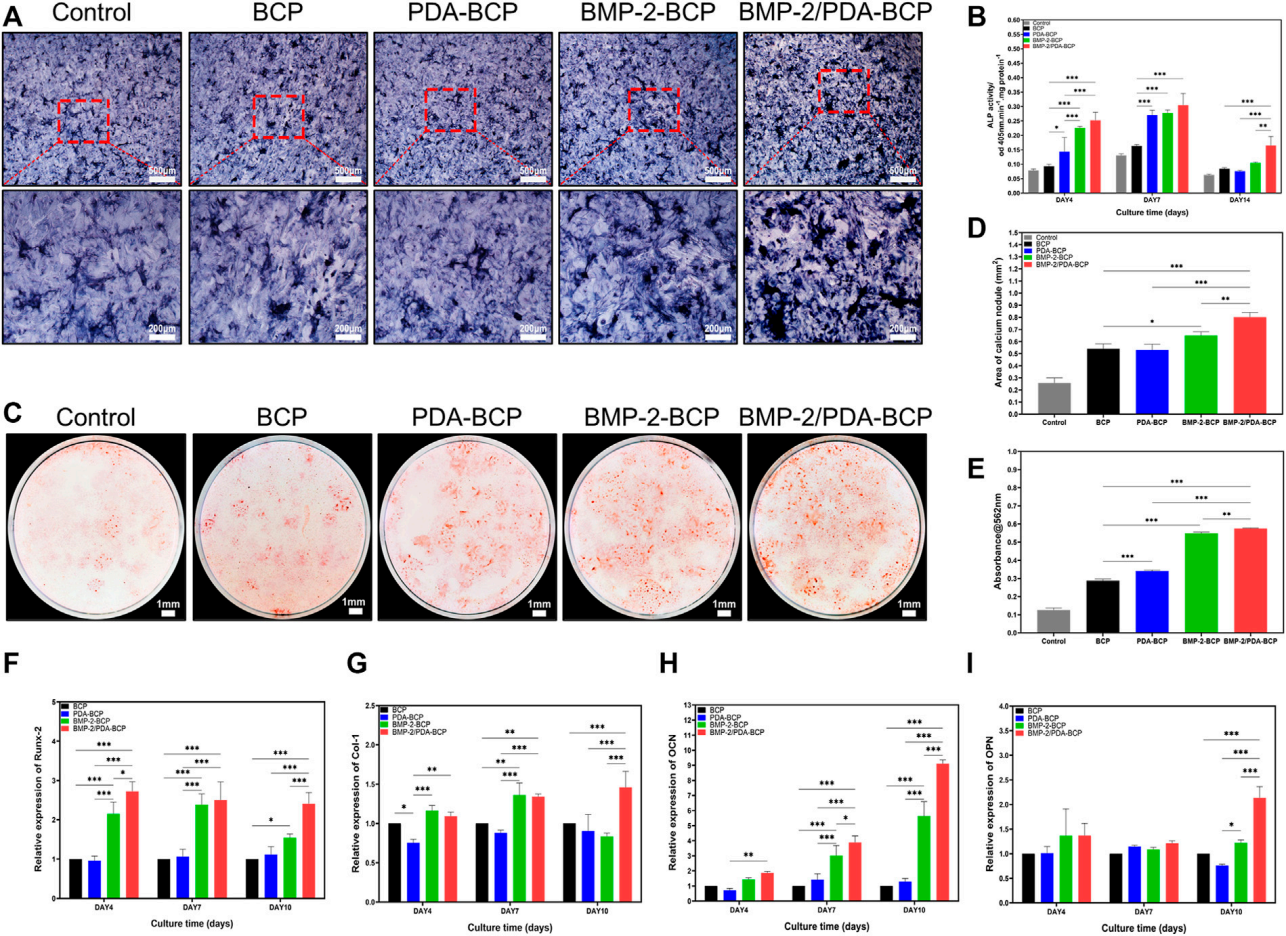
Effect of scaffolds on the osteogenic differentiation of BMSCs *in vitro*. **(A)** Representative microscopic images of ALP staining results for BMSCs after cultivation with scaffolds for 10 d. Scale bars = 500 μm, 200 μm. **(B)** The ALP activity was normalized by the total cell protein (OD 560 nm) of each sample at Days 4, 7, and 14 after seeding. **(C)** Representative digital images of alizarin red S staining of cells cultured in osteoinductive conditional medium for 21 d. Scale bars = 1 mm. **(D)** Quantification of alizarin red S staining area on various scaffolds from Figure 5C by ImageJ software. **(E)** Results of quantifying the amount of alizarin red S. Data are presented as the mean ± S.D. **p < 0.01, ***p < 0.001. qRT-PCR of osteogenic gene expression in BMSCs at 4, 7, and 10 d after seeding onto BCP, PDA-BCP, BMP-2-BCP, and BMP-2/PDA-BCP scaffolds under osteoinductive conditions, including **(F)** Runx-2, **(G)** Col-1, **(H)** OCN, and **(I)** OPN. *p < 0.05, **p < 0.01 and ***p < 0.001.

ALP staining and ALP activity assay were used to monitor osteogenic differentiation of BMSCs on the scaffolds. The most intense ALP staining was observed in BMP-2/PDA-BCP scaffold among all the groups, followed by BMP-2-BCP scaffold (Figure 5A). As shown in Figure 5B, BMSCs on BMP-2/PDA-BCP scaffold exhibited the highest ALP activity. Also, cells on BMP-2-BCP and PDA-BCP scaffolds exhibited higher ALP activity compared with the bare BCP scaffold. The mineralization of BMSCs was examined by alizarin red staining of calcium nodules after 21 days of culture (Figure 5C). It was found that the calcium nodule area of the BMP-2/PDA-BCP group was the largest, followed by the BMP-2-BCP group (Figure 5D). The OD values of dissolved alizarin red staining was well agreed with the results of calcium nodules counting (Figure 5E).

The qRT-PCR results of relative mRNA expression levels of osteogenic genes were displayed in Figures 5F–I. Generally, the BMP-2/PDA-BCP scaffold augmented the highest expression of OCN, Runx-2, and Col-1 on Day 4, 7, and 10. In addition, the BMP-2-BCP scaffold exhibited significant higher expression level of Runx-2 and OCN compared with PDA-BCP and BCP scaffolds on all time intervals. Also, the immunofluorescence staining of osteogenic-related proteins (OCN and OPN) (Supplementary Figure S7) was the most pronounced on the BMP-2/PDA-BCP scaffold over the other groups, followed by the BMP-2-BCP scaffold.

### 3.3 *In vivo* Biocompatibility and Osteogenesis of BMP-2/PDA-BCP Scaffold

**FIGURE 6:**
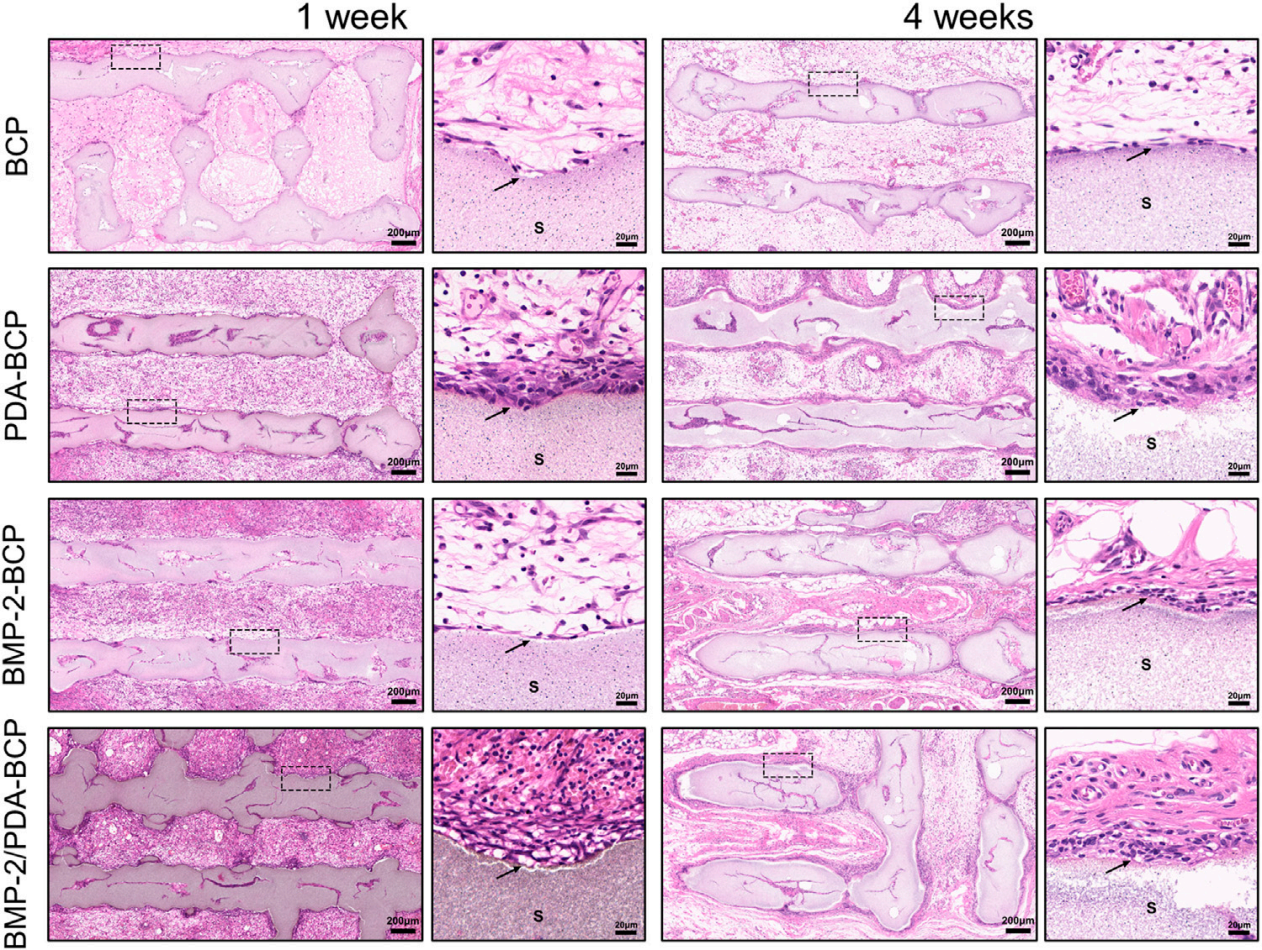
Histological evaluation of the precursor cells infiltration in the ectopic osteogenes model of SD rats after 1 and 4 weeks. Original magnification ×50, ×500; scale bar = 200 μm, 20 μm. Black arrows: cell adhesion layer, S: scaffolds.

The results of subcutaneous implantation showed that all the scaffolds displayed good biocompatibility *in vivo*, without obvious inflammatory cells infiltration (Figure 6). Also, no obvious ectopic bone tissue formation could be found within all the scaffolds. Notably, an interesting phenomenon can be observed that there are several layers of fibroblast-like cells formed adjacent to the scaffolds with PDA nanocoating on Week one and Week 4. Comparatively, a thinner cell layer formed alongside the BMP-2/BCP scaffold at 4 weeks, while the bare BCP scaffold did not show such phenomenon.

**FIGURE 7:**
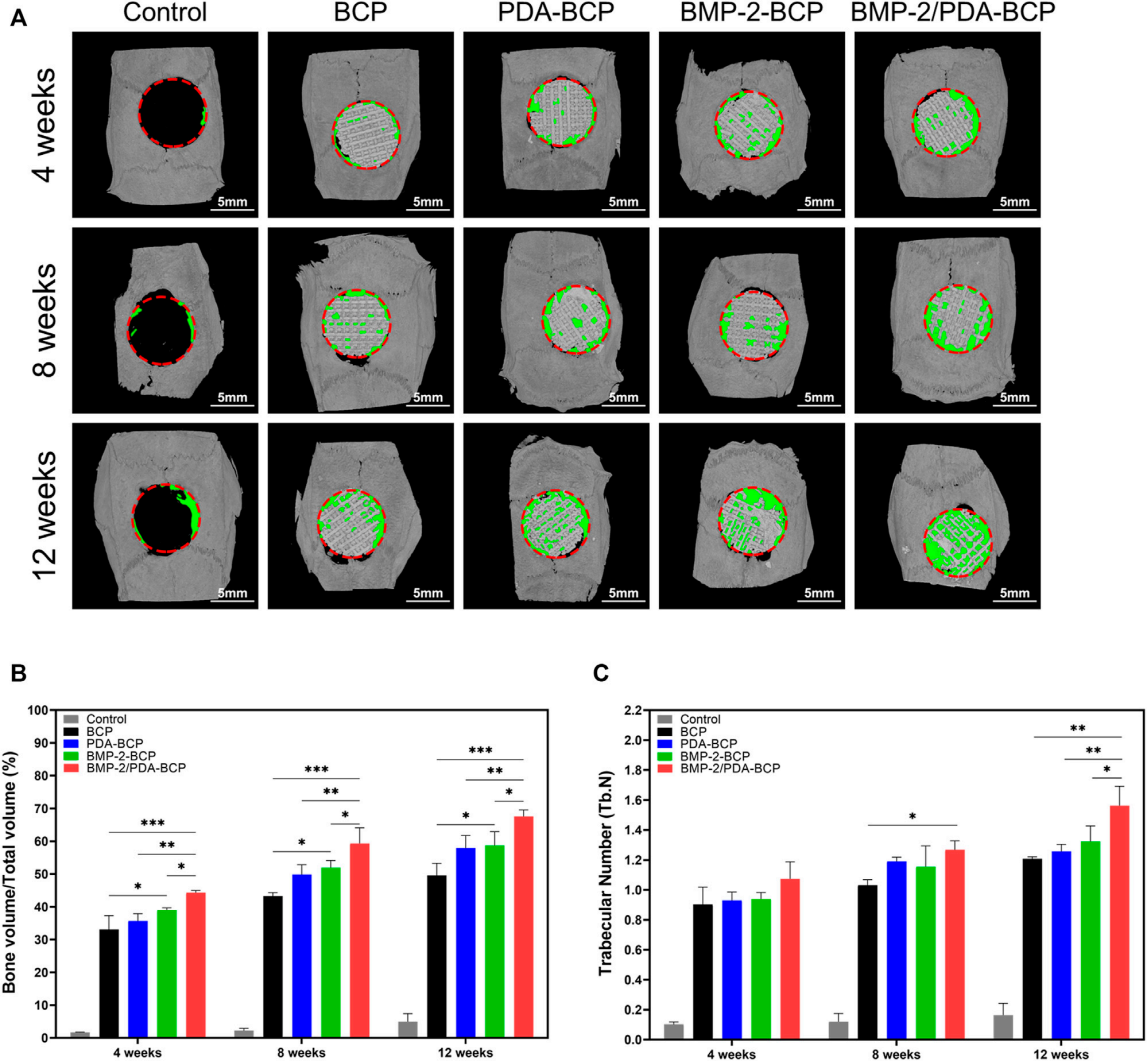
Micro-CT analysis of the effects of various scaffolds on new bone formation in the critical-size bone defect model of SD rats. **(A)** Three-dimensional reconstruction of micro-CT images of the various scaffolds implanted in the rat calvarium at 4, 8, and 12 weeks. Scale bars = 5 mm. **(B)** Regenerated bone volumes on the various scaffolds were quantified as bone volume divided by total volume (BV/TV). *p < 0.05, **p < 0.01, and ***p < 0.001. **(C)** The number of bone trabeculae were analyzed by software. *p < 0.05, ***p < 0.001.

3D micro-CT data of calvarial bone defect repair in SD rats are presented in Figure 7A. In all four groups, the newly formed bone increased in volume from 4 to 12 weeks. The most amount of new bone was generated in the BMP-2/PDA-BCP group, followed by the BMP-2-BCP group. On Week 12, bone volume/total volume (BV/TV) and trabecular number (Tb.N) values of the BMP-2/PDA-BCP group were the largest among all the groups (Figures 7B,C).

**FIGURE 8:**
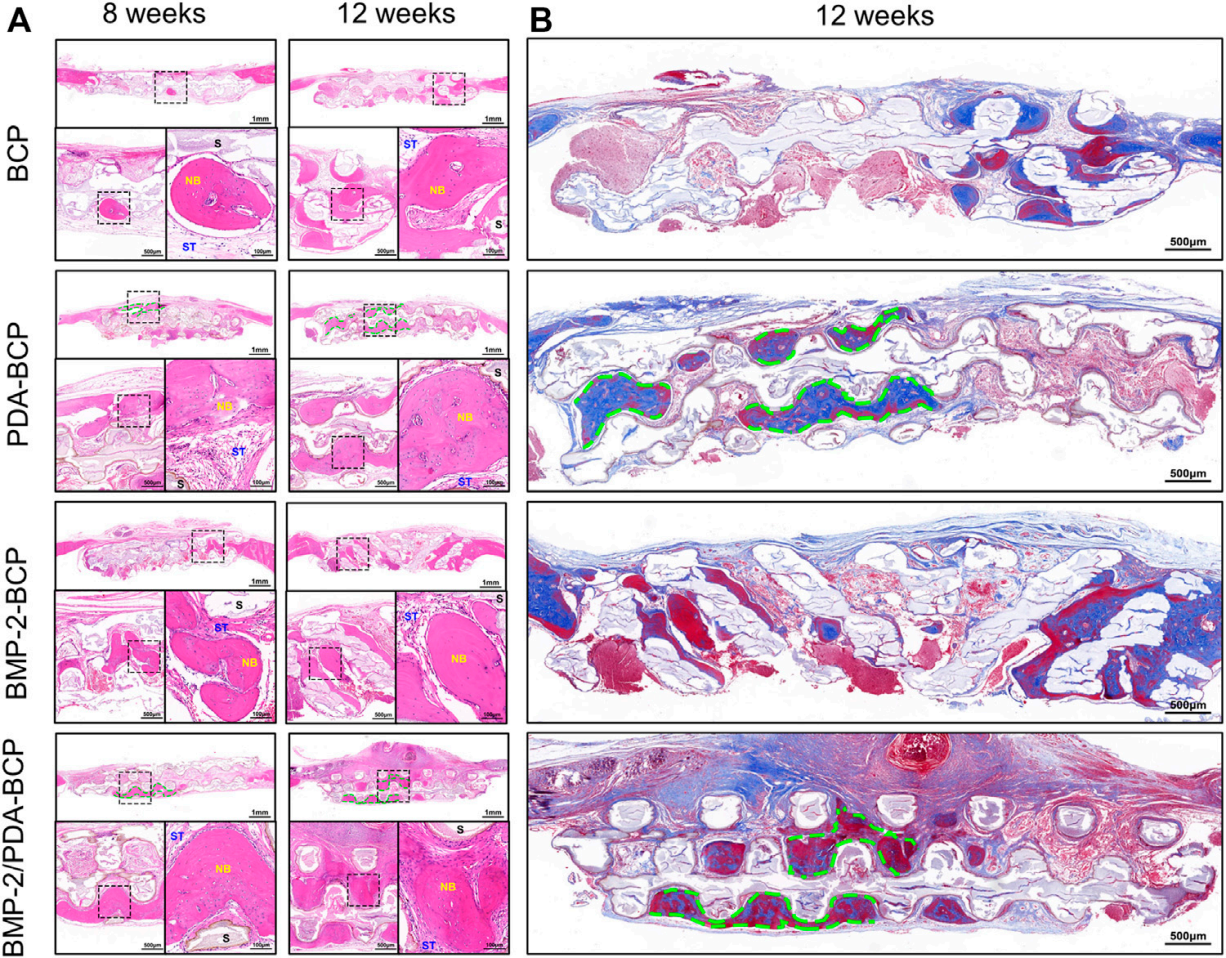
Histological evaluation of new bone formation in the critical-size bone defect model of SD rats after 8 and 12 weeks. **(A)** Representative full image of H&E staining of cranial bone defects. Original magnification: 10×, 30×, 100×; scale bar = 1 mm, 500 μm, 100 μm. Green dotted line: continuous new bones; NB: newly formed bone; ST: soft tissue; S: scaffolds. **(B)** Full images of Masson’s trichrome staining of decalcified bone in the defect area. Original magnification ×20; scale bar = 500 μm.

H&E staining revealed progressive bone formation in four groups from 8 to 12 weeks (Figure 8A). The newly formed bone tissues seemed the largest in amount for the BMP-2/PDA-BCP group. Notably, continuous new bone generated alongside the struct interspace (marked with green dotted lines) of PDA-BCP and BMP-2/PDA-BCP scaffolds, while such phenomenon cannot be observed in other scaffolds. In addition, the new bone tissue of the BMP-2-BCP and PDA-BCP scaffolds was more than the bare BCP scaffold. Consistent with H&E images, Masson staining images of 12 weeks showed that the BMP-2/PDA-BCP group obtained the greatest new bone ingrowth (Figure 8B), which is further proved by statistical result of new bone area (Supplementary Figure S8A). Similarly, continuous new bones could also be observed alongside the structure interspace (marked with green dotted lines) of PDA-BCP and BMP-2/PDA-BCP scaffolds. As shown in Supplementary Figure S8B, immunohistochemical images showed that the BMP-2/PDA-BCP group exhibited the highest expression levels of OCN, followed by the BMP-2-BCP group. The positively stained region of OCN was distributed in the extracellular matrix, especially in bone tissue.

## 4 Discussion

Owing to its high accuracy and fast speed, DLP-based 3D printing shows great application potential in fabrication of bioceramic bone scaffold for bone defects with small size and complex structure. But until now, comprehensive design and systematic biological evaluation about DLP-printed bioceramic scaffold are relatively less reported. In this study, we successfully fabricated 3D interconnected porous BCP scaffolds by DLP-based printing technique and further obtained a self-assembled Ca-P/PDA nanolayer, which can sustainedly release BMP-2 protein. Through systematic physicochemical characterization, BMSCs evaluation, and *in vivo* experiments, the BMP-2/PDA-BCP scaffold exhibited excellent biocompatibility and enhancement effect on accelerating bone regeneration in continuous new bones formation.

3D printing based on the DLP technique is one of the attractive technologies suitable for preparation of high-performance porous bioceramics. This technique has the advantages of fast-forming speed, template-free, high-tunability, and high precision. It allows adequate control of accurate and versatile structure, which is relatively challenging for other fabrication methods (Roseti et al., 2017; Zeng et al., 2018). The size error between the designed model and the actual printing scaffolds can be guaranteed by setting a compensation error value induced by shrinking rates (Supplementary Figure S1). The DLP-printed BCP scaffold had no obvious deformation after sintering and the high-interconnected pores could enhance mass transfer and benefit cellular penetration and tissue ingrowth (Tang et al., 2019). Additionally, the PDA-modification had no adverse effect on the mechanical strength of the scaffold. The compressive strengths of the PDA-BCP scaffolds were comparable with that of cancellous bone and thus were sufficient for non-load bearing bone regeneration (Morgan et al., 2018).

The SEM, FTIR, and EDS results together demonstrated the formation of Ca-P/PDA composite nanolayer on the BCP scaffold surfaces, which is consistent with the study published by Wu et al. and the formation mechanism was also clarified in that literature (Wu et al., 2014). Briefly, the formation mechanism of a Ca-P/PDA composite nanolayer involved two steps: 1) the addition of DA to Tris–HCl solution decreases the pH value and accelerates Ca and P ionic dissolution from the crystal boundaries of BCP ceramics; and 2) DA is polymerized to form self-assembled PDA film and, at the same time, Ca-P nanoparticles are mineralized with the assistance of PDA, in which the formation of PDA occurs simultaneously with Ca-P mineralization, and eventually a self-assembled Ca-P/PDA nanolayer forms. In addition, the EDS results (Supplementary Figure S4) revealed that the Ca/P ratio of the Ca-P deposits assembled on the PDA-BCP scaffold (Ca/P: 1.607–1.640) are higher than that of the pristine BCP scaffold (Ca/P: 1.538). Thus, it is speculated that the unstable *ß*-TCP phase (Ca/P: 1.5) in BCP scaffold transforms into more stable HA phase (Ca/P: 1.67) (Lee et al., 2014).

The surface roughness values and the wettability of the scaffolds changed after PDA modification. The Ra values decreased after PDA modification and decreased with the dopamine concentration. It is possible because the Ca-P/PDA deposits may fill some microscale cavities in the scaffold. In contrast to our observations, surface roughness increase due to PDA modification has been reported in previous literature (Wu et al., 2014). However, the PDA coating was deposited on *ß*-TCP discs with relatively smoother surfaces. Superhydrophilicity were mainly related to the Ca-P/PDA nanolayer, which contained abundant hydrophilic groups (NH2- and OH-) as well as nanostructures (Lynge et al., 2015; Qiu et al., 2018). Most studies have found that hydrophilic surfaces tend to enhance the early stages of osteoblast adhesion, proliferation, differentiation, and bone mineralization compared to hydrophobic surfaces (Wang et al., 2019; Lee et al., 2020; Wu et al., 2020). In addition, the dissolution rates of Ca and P ions from the PDA-BCP scaffold were slowed down, probably due to the physical diffusion barrier of the PDA coating and the promotion of the secondary mineralization induced by PDA layer (Kaushik et al., 2020). Additionally, the long-term (35 d) sustained-release of BMP-2 was achieved through PDA layer immobilization. Compared with physical adsorption and other chemical conjunction techniques, the PDA chemistry is rather cost-effective, versatile, and suitable for growth factors delivery of 3D porous scaffolds (Cheng et al., 2019). It is considered that primary amine group of BMP-2 can covalently and non-covalently bind to the catechol/quinone groups in the PDA (Hauser et al., 2020).

Taken together of all the *in vitro* and *in vivo* results (Figures 4–8), we can generally conclude that the PDA-BCP scaffolds have advantages compared with BCP scaffold in BMSCs viability, adhesion, and proliferation, and new bone formation. These improvements should be related to the comprehensive property enhancement induced by PDA coating, including nanostructures, superhydrophilicity, and chemical groups of PDA. Previous studies have considered that PDA coating will increase serum proteins adsorption, which further favor cell adhesion (Meng et al., 2020; Wang et al., 2020). Jo et al. (2013) and Lee et al. (2016) demonstrated that PCL scaffolds modified with PDA facilitates cell proliferation and migration. In addition, different from PDA coating on polymer scaffold, the as-prepared Ca-P/PDA coating also exhibited a nano-sized porous topography and it has been widely accepted that hierarchical porous structure is beneficial for BMSCs behaviors and *in vivo* osteogenesis (Xu et al., 2016).

Notably, cell layers were densely distributed alongside the PDA-modified scaffolds in the subcutaneous implantation experiments. The possible explanation is that the PDA coating improved cell adhesion and proliferation, which has been demonstrated by *in vitro* cell experiments. Additionally, continuous new bones formed alongside the struct interspace for PDA-modified scaffolds in rat cranial defects. These two phenomena might have close association. To the best of our knowledge, this is the first report on these phenomena for PDA-coated scaffold, and the underlying mechanism needs to be studied in the future. The BMP-2/PDA-BCP scaffold possessed the best osteogenic performances, which must be ascribed to the synergistic effect of the 3D structure of the scaffold, the BCP bioceramic, Ca-P/PDA nanocoating, and sustained-release of BMP-2. These factors together composed a favorable osteogenic microenviroment. Thanks to the DLP-based 3D printing technology, the porous BCP ceramic scaffold possessed a macroporous structure with interconnected pores, which is beneficial for oxygen and nutrient transportation, as well as bone tissue formation and blood vessels ingrowth. The chemical and phase composition of BCP bioceramics are similar with natural bone, which is crucial for its bioactivity and osteoconductivity. As mentioned above, the PDA modification endowed the scaffold with nano-sized topography, superhydrophilicity, and chemical groups of PDA, which facilitate BMSCs viability, adhesion, and proliferation. BMP-2 is currently the only Food and Drug Administration (FDA)-approved osteoinductive growth factor used as a bone graft substitute. Additionally, the good “secondary reaction” characteristics of PDA coating enable it to deliver BMP-2 effectively and release slowly *in vivo*, continuously acting on the osteogenic site, further enhancing the osteogenic ability of the scaffold. Collectively, these factors play a synergistic and positive role in the change of local microenvironment which can facilitate bone regeneration. We plan to carry out research on DLP-based 3D printed scaffold for repairing small-sized maxillofacial bone defects in large animal models for future transformation.

## 5 Conclusion

Porous DLP-printed BCP bioceramic scaffolds coated with polydopamine/BMP-2 were successfully fabricated. The scaffold exhibits an interconnected porous structure with a pore size of ∼570 μm and acceptable compressive strength (∼3.6 MPa) that matched with load-free cancellous bone. Additionally, a uniform Ca-P/PDA nanocoating was formed on the whole external and internal surface of the scaffold, which endowed the scaffold with superhydrophilicity and BMP-2 delivery and sustained-release abilities.

Furthermore, BMSCs showed improved cell viability, adhesion, proliferation, osteogenic differentiation, and mineralization on BMP-2/PDA-BCP scaffolds compared with bare BCP scaffold. *In vivo* results of subcutaneous implantation and cranial defect repair on SD rats demonstrated that the PDA coating induced cell aggregation nearby the coating and continuous lamellar new bone formation within the scaffold. Taken together, this study provided a promising strategy to fabricate bone substitute scaffolds for enhanced bone regeneration for bone defects in demand for high precision and small size.

## Data Availability

The original contributions presented in the study are publicly available. This data can be found here: https://www.ncbi.nlm.nih.gov/sra/, PRJNA791405.
