# Supplementary figures and images for: Preparation of BMP-2/PDA-BCP Bioceramic Scaffold by DLP 3D Printing and its Ability for Inducing Continuous Bone Formation
Source: Front Bioeng Biotechnol. 2022 Apr 6;10:854693. doi: 10.3389/fbioe.2022.854693 (PMC9019734; doi:10.3389/fbioe.2022.854693)

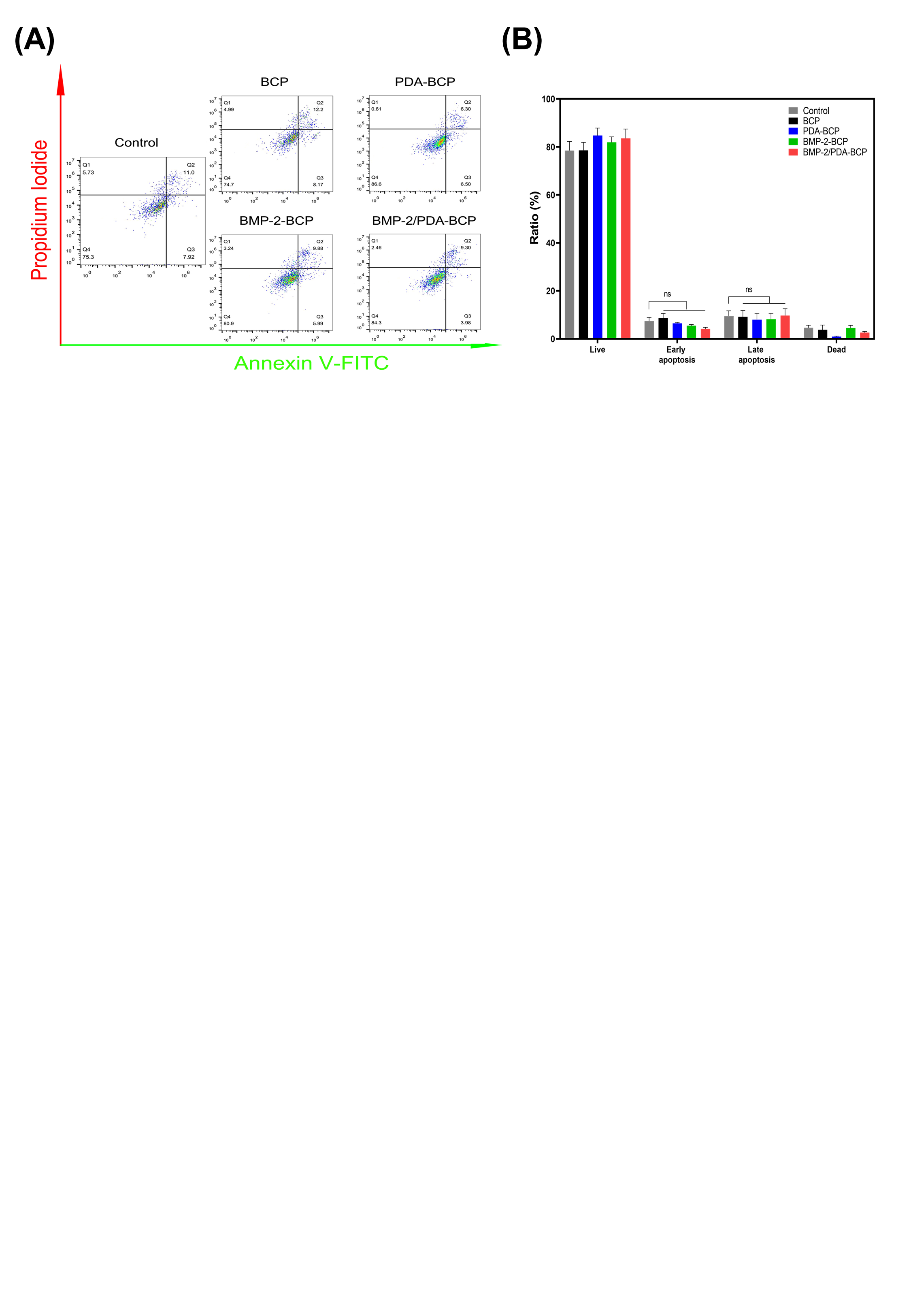

Supplement: Supplementary file 1 [file Image6.tif]

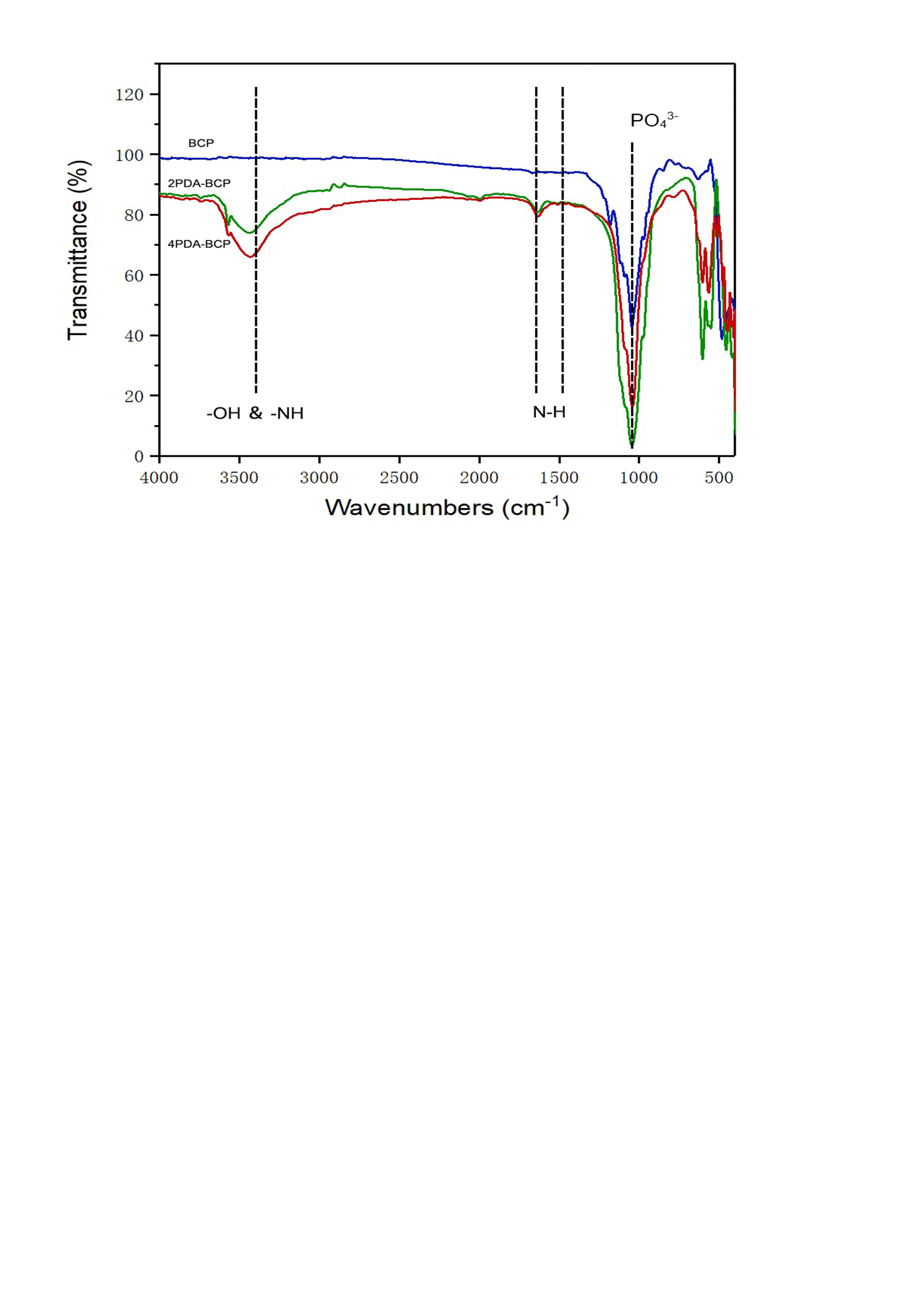

Supplement: Supplementary file 2 [file Image3.tif]

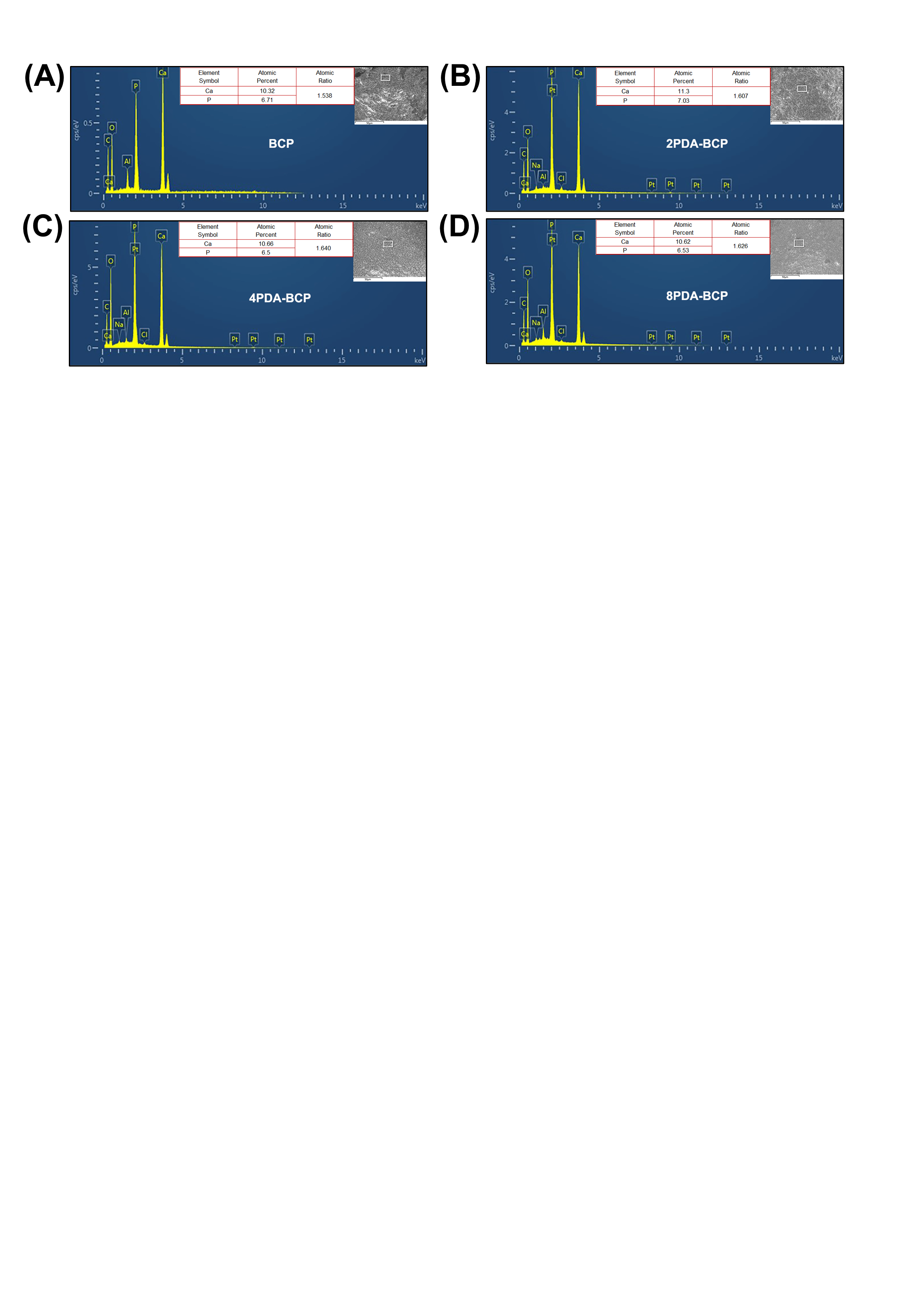

Supplement: Supplementary file 3 [file Image4.tif]

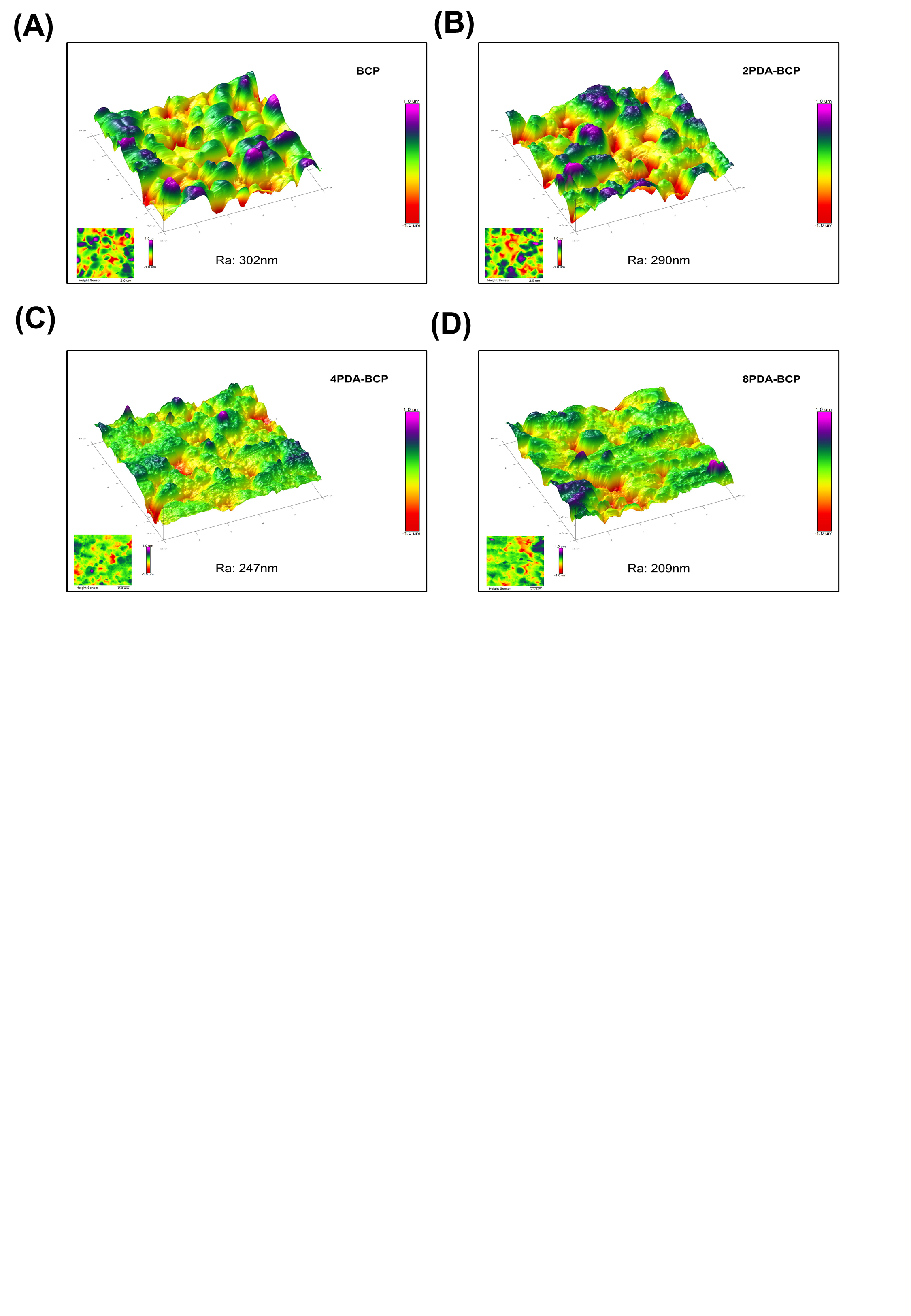

Supplement: Supplementary file 4 [file Image2.tif]

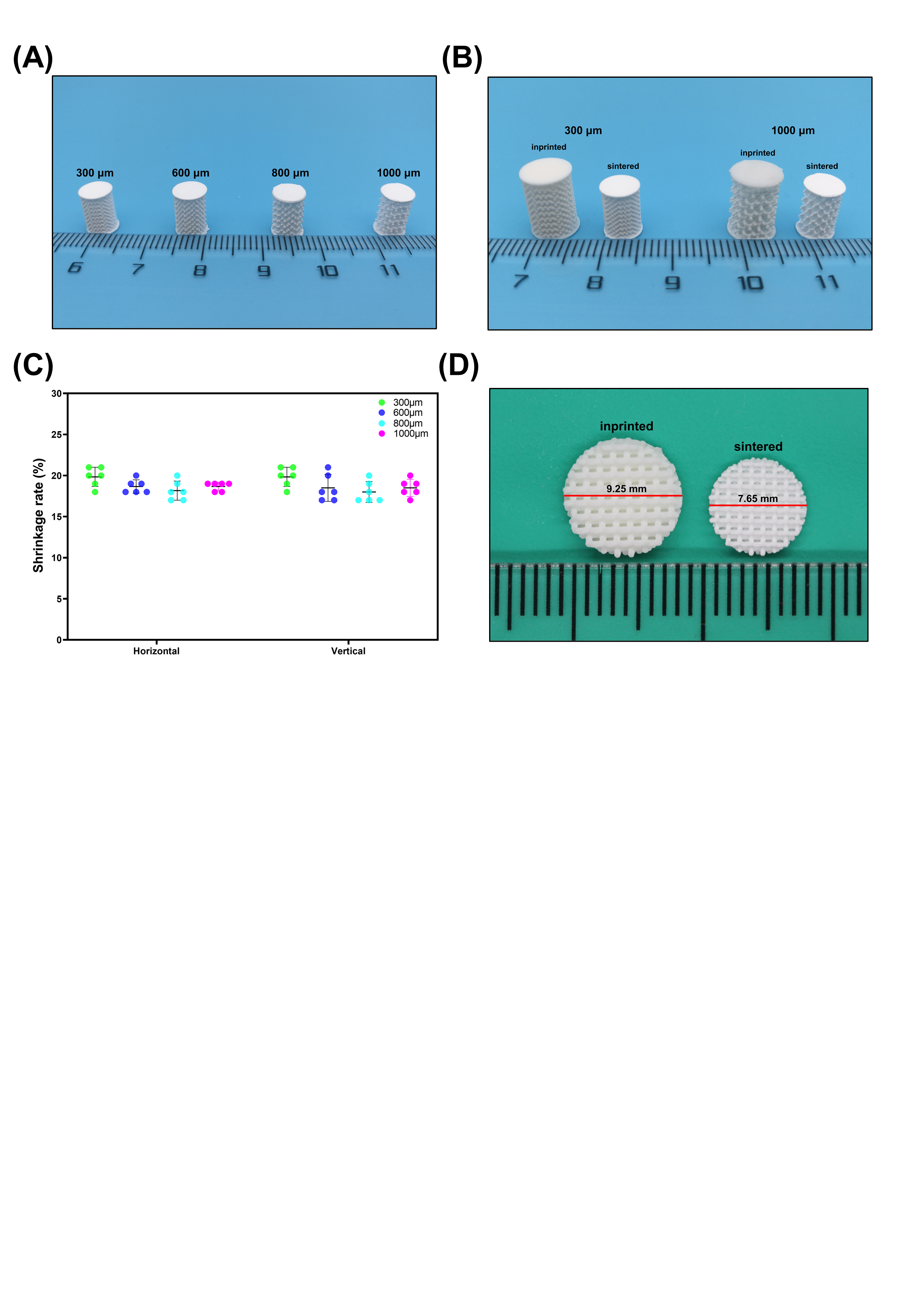

Supplement: Supplementary file 5 [file Image1.tif]

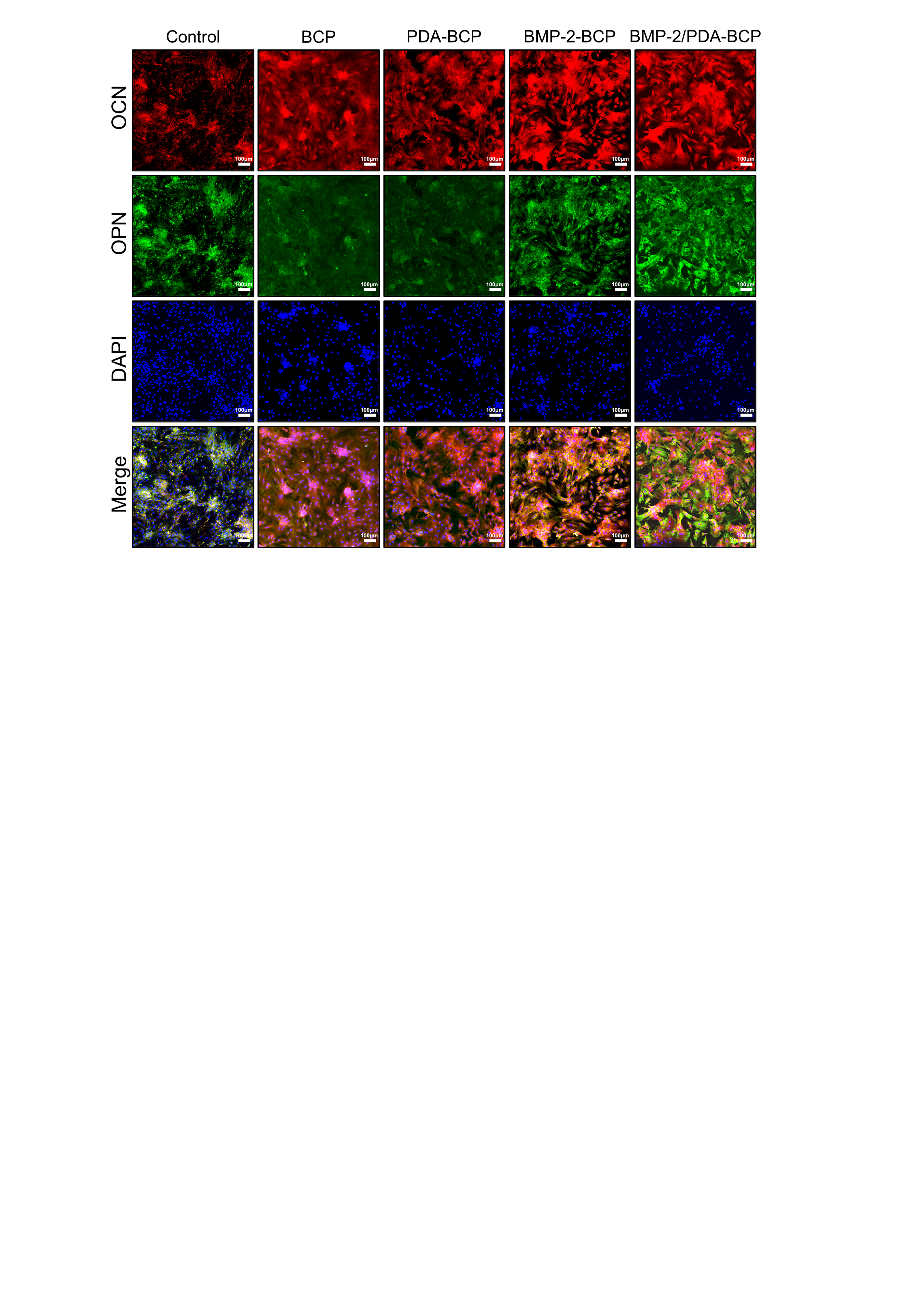

Supplement: Supplementary file 6 [file Image7.tif]

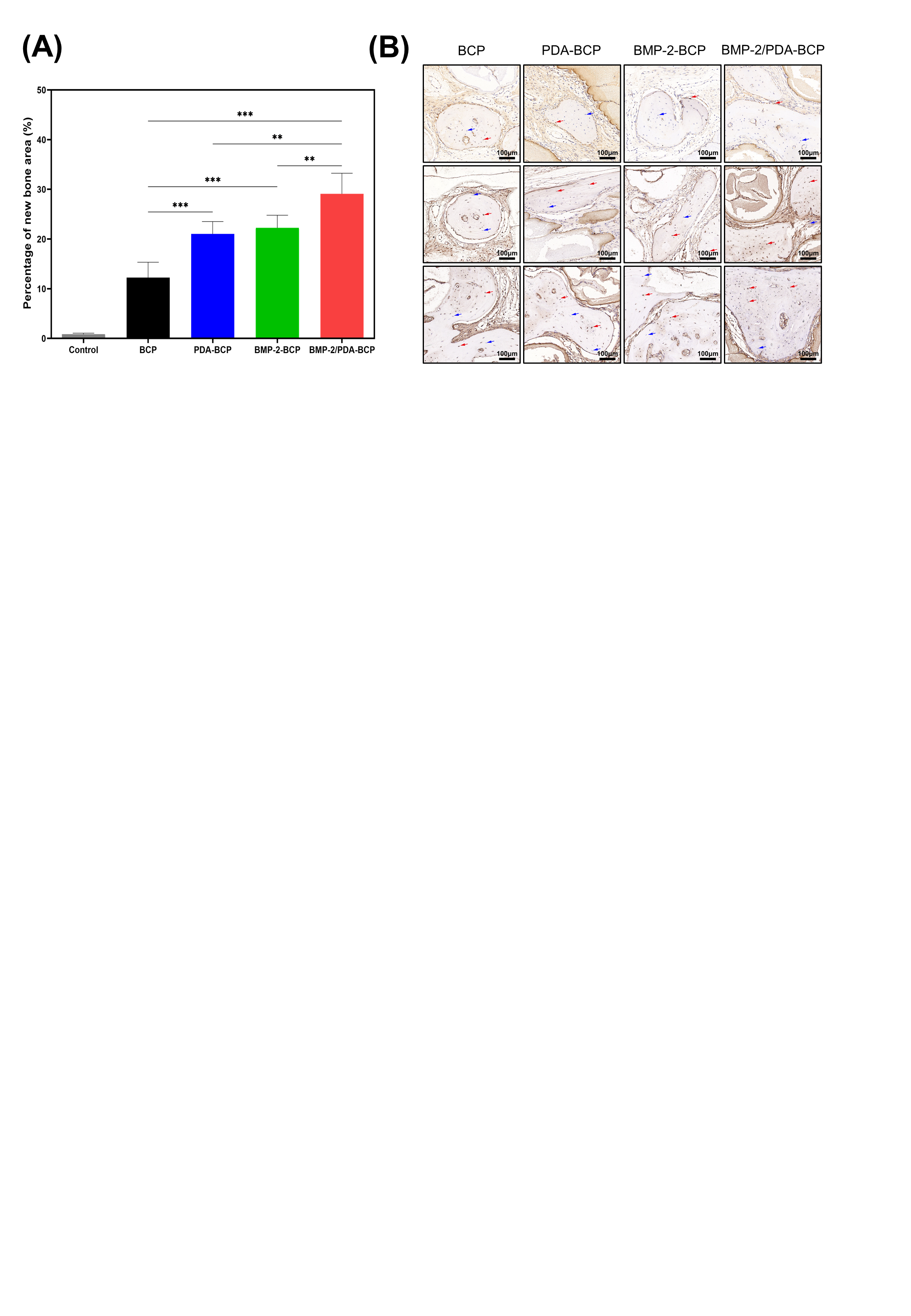

Supplement: Supplementary file 7 [file Image8.tif]

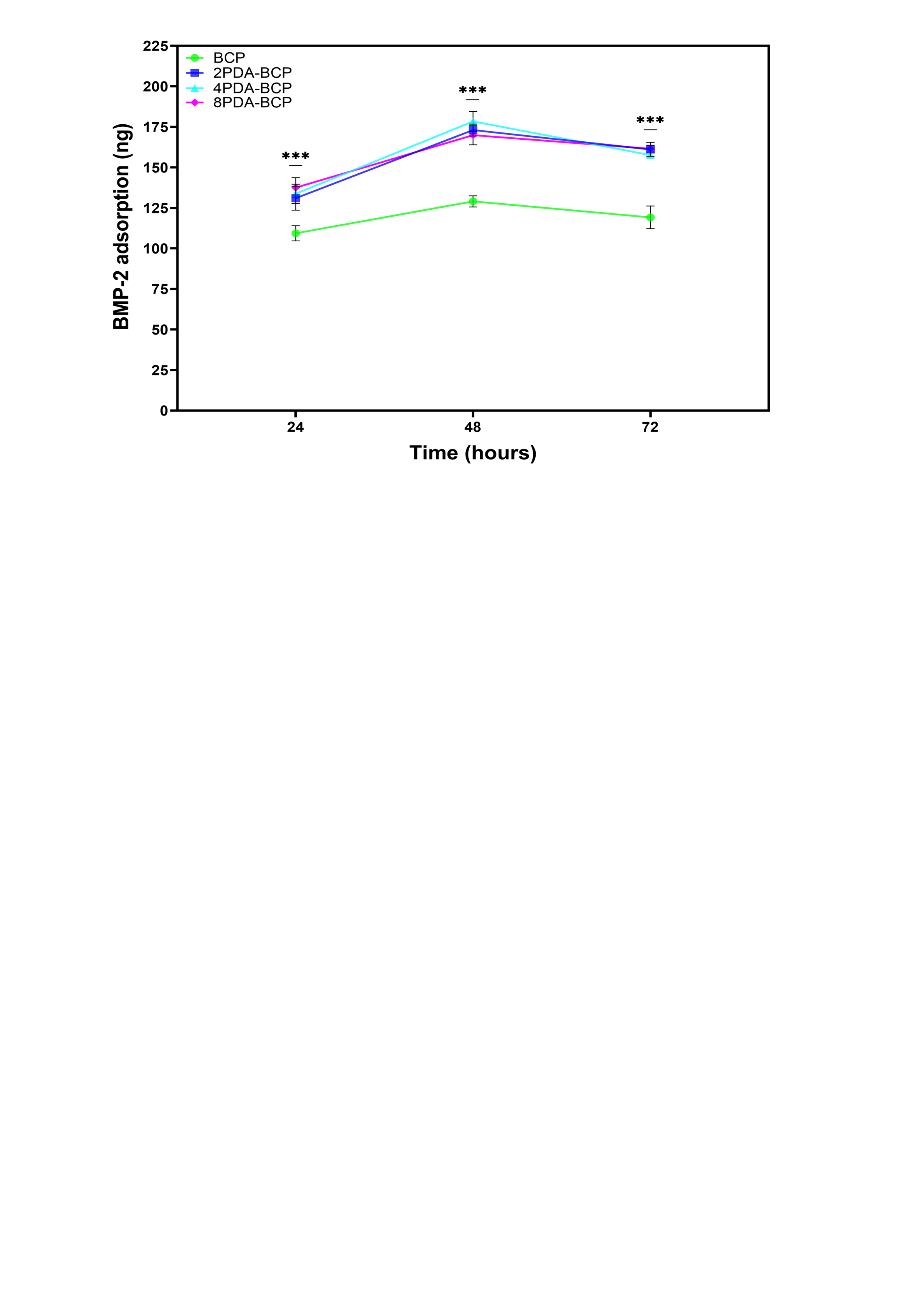

Supplement: Supplementary file 8 [file Image5.tif]
