# Supplementary material for: Preparation of BMP-2/PDA-BCP Bioceramic Scaffold by DLP 3D Printing and its Ability for Inducing Continuous Bone Formation
Source: Front Bioeng Biotechnol. 2022 Apr 6;10:854693. doi: 10.3389/fbioe.2022.854693 (PMC9019734; doi:10.3389/fbioe.2022.854693)
Supplement: Supplementary file 9 [file DataSheet1.docx]

# Supplementary Figure captions

## Supplementary Figure 1. Shrinkage of the scaffolds after sintering.

## Supplementary Figure 2. 3D and 2D images of the surface morphology of scaffolds. (A) BCP; (B) 2PDA-BCP; (C) 4PDA-BCP; (D) 8PDA-BCP. The images show the surface morphology were detected by Atomic Force Microscope (AFM).

## Supplementary Figure 3. FTIR of BCP scaffold and polydopamine coating on BCP scaffolds(2PDA-BCP, 4PDA-BCP).

## Supplementary Figure 4. Energy-dispersive spectrometry (EDS) analysis of the elemental compositions of BCP (A) and 2, 4, 8mg/mL PDA-BCP scaffolds(B, C, D), inserted tables showing the ratio of Ca, P ion, respectively.

## Supplementary Figure 5. Evaluation of protein loading capacity of scaffolds. The amount of grafting BMP-2 for variety of scaffolds at different conditions.

## Supplementary Figure 6. Cellular compatibility of BMSCs on scaffolds. (A) The apoptosis level of BMSCs incubated with scaffolds was measured using the annexin V-FITC/PI assay. (B) Proportions of live and apoptotic BMSCs at 7 days after seeding on scaffolds, as assessed using flow cytometry.

## Supplementary Figure 7. Representative immunofluorescent images showing the effect of different scaffolds on the protein expression of OCN and OPN after culturing for 14 days. OCN are labeled by red fluorescence, while OPN are marked by green fluorescence, cell nuclei were counterstained with DAPI (blue fluorescence).

## Supplementary Figure 8. (A) Quantification of newly formed bone from Fig. 8(B). The data are presented as the mean ± SD, **p<0.01, ***p<0.001. (B) Immunohistochemistry showing OCN expression in the cranial bone defect. Red arrows: positively stained cells, blue arrows: negatively stained cells. Original magnification 100×; Scale bar = 100μm.
